# Synonymous Substitution Rates Predict HIV Disease Progression as a Result of Underlying Replication Dynamics

**DOI:** 10.1371/journal.pcbi.0030029

**Published:** 2007-02-23

**Authors:** Philippe Lemey, Sergei L Kosakovsky Pond, Alexei J Drummond, Oliver G Pybus, Beth Shapiro, Helena Barroso, Nuno Taveira, Andrew Rambaut

**Affiliations:** 1 Department of Zoology, University of Oxford, Oxford, United Kingdom; 2 Department of Pathology, University of California San Diego, La Jolla, United States of America; 3 Department of Computer Science, University of Auckland, Auckland, New Zealand; 4 Centro de Patogénese Molecular, Faculdade de Farmácia de Lisboa, Lisbon, Portugal; 5 Instituto Superior de Ciências da Saúde Egas Moniz, Lisbon, Portugal; 6 Institute of Evolutionary Biology, University of Edinburgh, Edinburgh, United Kingdom; University of Auckland, Australia

## Abstract

Upon HIV transmission, some patients develop AIDS in only a few months, while others remain disease free for 20 or more years. This variation in the rate of disease progression is poorly understood and has been attributed to host genetics, host immune responses, co-infection, viral genetics, and adaptation. Here, we develop a new “relaxed-clock” phylogenetic method to estimate absolute rates of synonymous and nonsynonymous substitution through time. We identify an unexpected association between the synonymous substitution rate of HIV and disease progression parameters. Since immune activation is the major determinant of HIV disease progression, we propose that this process can also determine viral generation times, by creating favourable conditions for HIV replication. These conclusions may apply more generally to HIV evolution, since we also observed an overall low synonymous substitution rate for HIV-2, which is known to be less pathogenic than HIV-1 and capable of tempering the detrimental effects of immune activation. Humoral immune responses, on the other hand, are the major determinant of nonsynonymous rate changes through time in the envelope gene, and our relaxed-clock estimates support a decrease in selective pressure as a consequence of immune system collapse.

## Author Summary

During the clinical course of HIV infection, an asymptomatic phase always precedes the acquired immunodeficiency syndrome (AIDS). The duration of this asymptomatic phase is highly variable among patients and reflects the rate at which the immune system gradually deteriorates. Although humoral and cell-mediated immune responses are mounted against HIV, continuous replication and adaptation allows the virus to escape host immune responses. To gain a better understanding of the role of viral evolution in disease progression, we developed a new computational technique that can estimate changes in the absolute rates of synonymous and nonsynonymous divergence through time from molecular sequences. Using this type of evolutionary inference, we have identified a previously unknown association between the “silent” evolutionary rate of HIV and the rate of disease progression in infected individuals. This finding demonstrates that cellular immune processes, which are already known to determine HIV pathogenesis, also determine viral replication rates and therefore impose important constraints on HIV evolution.

## Introduction

Although the clinical course of HIV infection is generally well-defined, there is considerable variability among patients in rates of disease progression. The highly variable asymptomatic phase, ranging from several months to more than 20 years, most likely reflects differences in the nature of the evolutionary arms race between the virus population and the host immune system. Both humoral and cell-mediated immune responses are mounted against the virus but are eventually defeated by HIV replication and adaptation. As part of this process, neutralizing antibodies (nAbs) exert a strong selective pressure on the HIV envelope gene *(env)* [1,2] but do not control viral replication, and nAb levels are not predictive of disease progression [3]. Cytotoxic T cell (CTL) responses play a more important protective role in HIV infection, and evidence has shown that partial control of HIV replication in vivo is temporally associated with the appearance of CTL responses [4] and that the rate of disease progression is strongly dependent on human leukocyte antigen (HLA) class I alleles [5,6]. Although CTLs may not be responsible for the majority of infected cell deaths, small differences in CTL killing rates could still be clinically relevant and alter the time of disease onset [7].

Unfortunately, the enormous potential for evolutionary change in HIV can counteract these host defense responses. High mutation and recombination rates coupled with rapid replication dynamics generate a genetically diverse viral population, enabling the infection of a large number of susceptible cells. Consequently, HIV is able to adapt readily to changing environmental conditions within each host. The envelope protein is able to evade nAb responses by accumulating multiple amino acid changes, especially in the hypervariable regions, while maintaining full functionality for viral cell entry. CTL responses, however, target epitopes in other viral genes (such as *gag* and *nef*) more strongly and/or more frequently, particularly during the initial stages of HIV infection [8–10]. Although HIV infection often results in the evolution of viral variants that escape CTL responses [11], recent evidence from transmission pair studies and in vitro growth rate studies suggests that some escape mutations might occur at the expense of viral fitness [12,13].

Although the immune response against HIV has become increasingly well-characterized, less is known about the role of viral evolution in disease progression, despite its importance to our understanding of virus–host dynamics during persistent infection. Comprehensive sampling of C2V3 *env* sequences from nine HIV-1 infected patients throughout the entire course of HIV-1 infection revealed consistent patterns of viral evolution: both genetic diversity of the viral population at a given time point and mean divergence from the founder strain increased approximately linearly during infection [14]. However, diversity peaked at roughly the same time that viruses using the CXCR4 coreceptors emerged, whilst divergence did not stabilize until close to the time of disease onset. Studies of viral diversity and divergence in HIV-1 patients with different disease progression rates often have conflicting results (e.g., [15,16]). Attempts to distinguish between adaptive and selectively neutral mutations have suggested that slower disease progression is associated with more positively selected sites and higher adaptation rates in *env* [17,18]. From an immunological perspective, however, it remains unclear whether such viral adaptation is the cause or consequence of variability in disease progression rates.

Although the physiological processes leading to CD4+ T cell depletion and AIDS are not clearly defined, it is widely accepted that persistent immune activation has a pivotal role in driving disease progression [reviewed in 19]. T cell activation is the strongest predictor of progression to AIDS in HIV-1 infected patients [20,21] and can determine the rate and continuity of viral replication [19]. Consequently, immune activation could also impose important constraints on viral generation times and HIV evolution. However, such effects have not been established through evolutionary analyses.

The ratio of nonsynonymous/synonymous substitution rates has proved useful in investigating molecular adaptation; however, changes in the *absolute* rates of nonsynonymous and synonymous substitution should provide greater insight [22]. Changes in synonymous substitution rates can reflect changes in generation time or mutation rate, while nonsynonymous rates can also be affected by changes in selective pressure and effective population size. Previous studies of HIV evolution have typically assumed that the rate of neutral or synonymous change (per month or year) is approximately constant among patients (e.g., [15,16]). This assumption may be inappropriate, as the rate depends on the average number of viral generations per unit time, which may vary. For example, viral replication rates can depend on the state of immune activation [23], viral strains may differ in their replicative ability in different environments [12], and average virus generation times can be affected by the dynamics of latently infected cells [24,25].

To investigate these issues in HIV infection, we developed a new statistical approach to estimate absolute rates of synonymous and nonsynonymous substitutions and to determine how those rates change through time. Our method extends previous relaxed-clock methods with codon model analysis and allows evolutionary rates to change in an uncorrelated fashion along branches in a genealogy [26]. By comparing evolutionary rates for specific branches in within-host HIV phylogenies, we correct for the potentially biasing effects of transient deleterious polymorphism. Using this approach, we investigate the relationships among viral substitution rates, disease progression, and host immune responses. Our results show that disease progression among patients is predicted by synonymous substitution rates, most likely reflecting different levels of persistent immune activation, while nonsynonymous rates evolve within patients as a consequence of changing antibody selective pressure.

## Results

**Figure 1:**
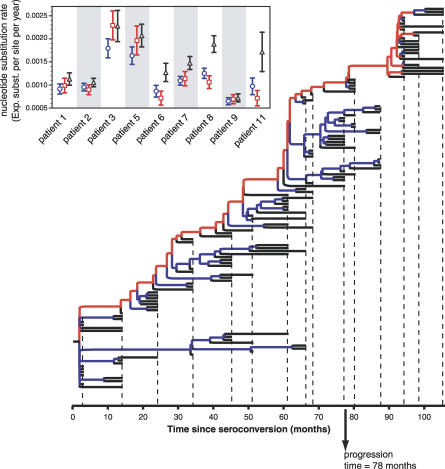
Internal, Backbone, and External Branches in a Within-Host HIV Genealogy, and Mean Nucleotide Substitution Rates for These Branches in Nine Longitudinally Sampled HIV-1 Patients To avoid the influence of deleterious mutations segregating to external branches in intrapatient HIV genealogies, we estimate mean substitution rates for the set of internal and backbone branches. These branch sets are depicted in color in the *maximum a posteriori* tree for “patient 1” obtained by Bayesian relaxed-clock inference [26] (backbone, red; internal, blue; external, black). The backbone represents the central trunk of trees shaped by rapid lineage turnover and can be defined phylogenetically (see Methods). Note that the set of internal branches also includes the backbone branches. Samples for each time point are indicated by the dotted line. Mean nucleotide substitution rates and their standard deviations on internal, external, and backbone branches are shown for all longitudinally sampled HIV-1 patients. The consistently higher substitution rate on external branches might be indicative of higher mutation load on these branches.

We estimated rates of nonsynonymous *(dN)* and synonymous *(dS)* divergence using longitudinal *env* sequence data from nine HIV-1 infected individuals [14,27]. The high mutation rate of HIV is expected to lead to a considerable deleterious mutation load in the viral population, such that the most recent mutations, segregating on external branches of HIV phylogenies, are likely to be deleterious [28,29]. This effect is present in each of the nine patients analyzed; mean substitution rates were consistently higher for external branches than for internal branches (Figure 1). Simulations indicated that such differences were not expected under a neutral model (Table S1). We also found no evidence supporting a link between high external rates and recombination rates for each time point (Table S2).

**Figure 2:**
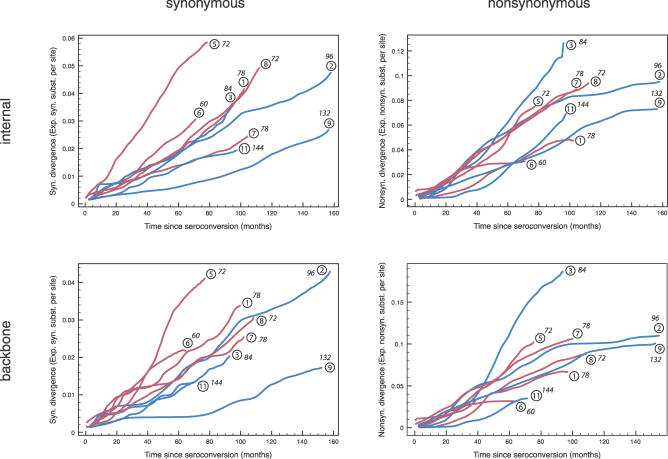
Mean Synonymous and Nonsynonymous Divergence for Internal and Backbone Branches over the Course of HIV Infection in Nine Individuals Patients with moderate and slow disease progression, categorized by progression time less than or greater than seven years [17], are shown in pink and blue, respectively. Individual patient numbers from Shankarappa et al. [14] are shown in circles, and progression times for each patient are indicated in italics.

Transient deleterious mutations do contribute to HIV evolution but not to the process of nucleotide substitution, since they are, most probably, rapidly purified [29]. Because inclusion of deleterious mutations could bias our estimates of divergence and absolute evolutionary rate, we estimated mean rates of divergence for two sets of branches: internal branches, and “backbone” branches representing the central lineage of the phylogeny that persists through time (Figure 1; see Methods for a phylogenetic definition of the backbone; specific details of the backbone, internal, and external branches for the other patients can be found in Figure S1). Estimates of synonymous and nonsynonymous divergence for internal and backbone branches are shown in Figure 2. When patients are classified as moderate or slow progressors (based on progression time, or the time it takes for the CD4+ T cell count to drop below 200 cells/μl; [17]), virus populations in slow progressors appear to accumulate synonymous substitutions more slowly than those in moderate progressors (Figure 2). This relationship is not apparent for nonsynonymous divergence.

**Figure 3:**
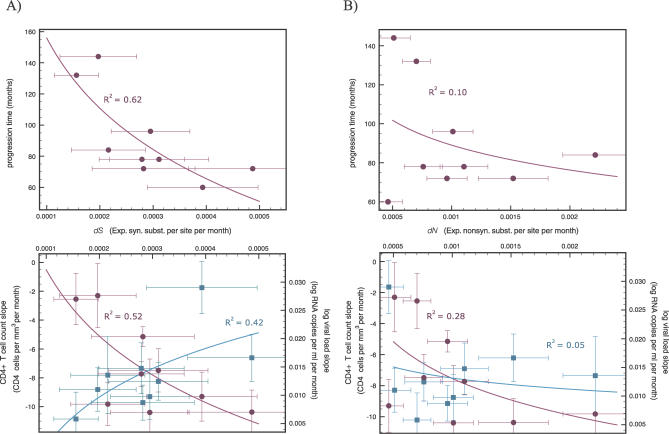
Scatter Plots of Mean Synonymous (A) and Nonsynonymous (B) Substitution Rates on Backbone Branches as a Function of Progression Time, CD4+ T Cell Count Slope, and Log Viral Load Slope Rates as a function of progression time and the slope of the CD4+ T cell count are shown in pink, while rates as a function of the log *VL* slope are shown in cyan. The progression time for patient 11 was set at the last sampling time (144 months), although the CD4+ count had not dropped below 200 cells/μl at that time [14,17]. The slope of the CD4+ T cell count and log *VL* were calculated based on linear regression of these parameters as a function of time. The error bars represent the standard errors of the estimates.

We investigated correlations between HIV divergence rates, estimated from the root of the within-host genealogies to progression time, and three continuous parameters that relate to disease progression: progression time, the rate of CD4+ T cell count change over time, and the rate of log viral load (log *VL*) change over time (Figure 3). The log of the backbone rate of synonymous divergence shows a strong negative correlation with both progression time (Pearson correlation coefficient *r* = −0.79, *p* = 0.011) and the change in CD4+ T cell count (*r* = −0.72, *p* = 0.028), and a moderate positive correlation with the change in log *VL* (*r* = 0.65, *p* = 0.059). No significant correlations were observed for nonsynonymous divergence rates (*r* = −0.32, *p* = 0.40 for progression time; *r* = −0.54, *p* = 0.135 for CD4+ T cell count change; *r* = −0.14, *p* = 0.58 for log *VL* change). Similar results were obtained when divergence rates were estimated from internal branches (Figure S2). In contrast to backbone and internal rates, no significant correlations were observed for both *dS* and *dN* rates on external branches. Similar results were also obtained when datasets were restricted to samples up to about 70 months after seroconversion (Figure S3), indicating that the differences in *dS* rate estimates could not be attributed to differences in time length of sampling. In general, backbone *dS* rates before progression time seem to show little temporal fluctuation in the trees since *dS* divergence accumulated in a linear fashion, with *R* ^2^ values close to 0.96, except for patient 9 (*R* ^2^ = 0.83). We also investigated the heterogeneity of synonymous and nonsynonymous substitution rates within the *env* C2V3 gene, because strong site-to-site variation in synonymous rates has the potential to bias *dS* estimates [30]. Although this analysis revealed very strong site-to-site variation in *dS* rates, the inferred rate distributions were nearly identical among all patients (Table S3). Finally, recombination rate estimates (Table S2) did not provide any evidence that recombination could be the cause of the differences between *dS* estimates.

**Figure 4:**
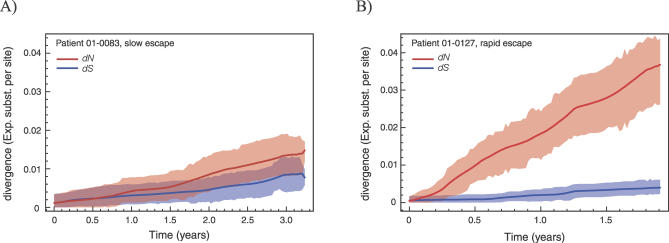
Internal Synonymous and Nonsynonymous Divergence in Two Patients with Distinct Phenotypic Escape from Neutralizing Antibodies Nonsynonymous and synonymous divergence is shown for complete *env* sequences serially sampled from patient 01–0083 (A), whose virus exhibited slow escape from neutralizing antibodies, and from patient 01–0127 (B), whose virus exhibited rapid escape from neutralizing antibodies [2]. The thick lines depict the mean divergence and the colored areas represent the 95% credibility intervals.

The variability in *dS* rates could reflect differences in either viral mutation rate or viral generation time, but only the latter provides a likely explanation for the correlation between *dS* rates and disease progression. However, viral generation time is also expected to affect *dN* rates to some extent. While we did not observe a significant correlation, this may be because *dN* rates are determined primarily by the strength of selection on viral mutations, rather than by the absolute rate at which those mutations are generated. Furthermore, most diversifying selection in *env* results from nAb responses [31], which are not expected to moderate replication rates and disease progression. To demonstrate this, we further analyzed *env* sequences sampled through time from two patients with markedly distinct rates of phenotypic escape from nAb responses [31] (patient 01–0083 and patient 01–0127; Figure 4). As in Frost et al. [2], we show that the virus that rapidly escaped nAb responses in patient 01–0127 accumulated nonsynonymous substitutions at a considerably higher rate on backbone branches, while synonymous divergence rates appear to be unaffected (similar plots were obtained for internal branches, unpublished data).

Because it has been shown that viral divergence stabilizes close to disease onset [14], we estimated mean divergence rates *prior* to the progression time in the analyses above. Two hypotheses have been proposed to explain this stabilization: reduced availability of target cells late in infection (the “cellular exhaustion” hypothesis), or reduced selective pressure because of deteriorating immune responses (the “immune relaxation” hypothesis) [32]. A recent statistical analysis provided support for the immune relaxation hypothesis by showing that nonsynonymous divergence stabilizes at about the same time as progression time, while synonymous divergence does not [32]. Our analysis provides further evidence that nonsynonymous divergence stabilizes in some patients and that this is less pronounced for synonymous divergence (Figure 2). Using the empirical relaxed-clock approach, we directly estimated *dN* and *dS* substitution rates before and after progression time (Table 1). These estimates indicate that *dN* is significantly lower after progression time both for internal and backbone branches (paired *t* test: *p* = 0.012 and *p* = 0.001, respectively; Wilcoxon signed rank test: *p* = 0.016 and *p* = 0.008, respectively), while there is no significant difference in *dS* before and after progression time (paired *t* test: *p* = 0.424 and *p* = 0.333, respectively; Wilcoxon signed rank test: *p* = 0.461 for internal and backbone branches).

**Table 1:**
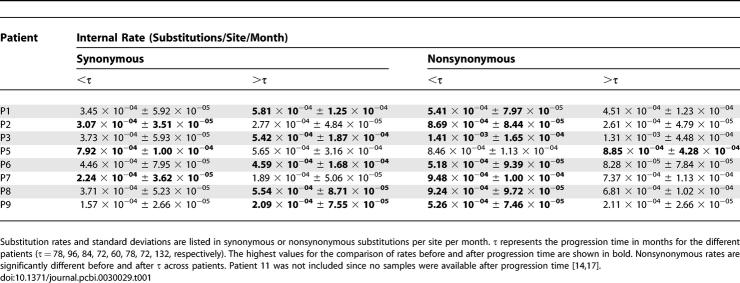
Mean Synonymous and Nonsynonymous Substitution Rates Before and After Disease Progression

**Table 1:**
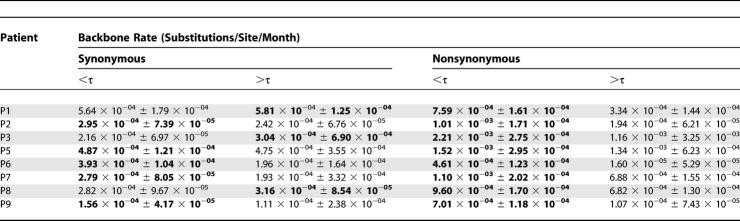
Extended.

As an extension to the analysis of closely related HIV-1 strains, we further explored differences among within-patient substitution rates for more divergent HIV lineages. Table 2 lists average *dS* and *dN* rates for HIV-1 subtype B infected patients, HIV-1 group O infections, and HIV-2 datasets. Although studies of HIV-1 group O infection are limited, no differences in disease progression between group O and group M infections have been observed [33]. HIV-2, on the other hand, is known to be less transmissible and less pathogenic than HIV-1 group M [34,35]. The two HIV-1 group O datasets had *dS* rates that were similar to the rates estimated for HIV-1 subtype B moderate progressors (Table 2). Clinical data for these HIV-1 group O patients indeed indicate progression times in the range of moderate progressors (60 and 70 months; [33,36]). In contrast, HIV-2 patients had *dS* rates that were more comparable with HIV-1 group M slow progressors (Table 2). The two published HIV-2 datasets (P7P8 and P9P10) represent cases of perinatal transmission only diagnosed in adulthood [37], as expected, given the slow nature of HIV-2 disease progression. HIV-2 *dN* rates are in the same range of HIV-1 *dN* rates in both moderate and slow progressors. Interestingly, the observation that group O viruses are characterized by a high *dN* is in agreement with a population-level study that identified more sites under positive selection in *env* for HIV-1 group O than for HIV-1 group M or HIV-2 [38].

**Table 2:**
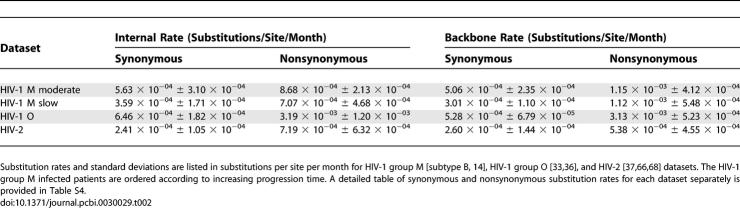
Mean Synonymous and Nonsynonymous Substitution Rates for Different HIV Variants

## Discussion

The relationship between HIV evolution and disease progression is central to our understanding of immune control and to vaccine design. Although much research effort has been focused on this issue, a clear and coherent picture of HIV evolutionary dynamics has yet to be presented. Here, we develop a novel computational approach to estimate the absolute rates of synonymous and nonsynonymous substitution during the course of HIV infection.

Our analyses reveal that slower progression to AIDS is strongly correlated with a slower rate of synonymous substitution, indicative of a slower replication rate and longer viral generation times. This reasoning assumes that synonymous substitutions are selectively neutral. Although synonymous mutations may influence fitness in viral populations, experimental exploration of fitness effects caused by single-nucleotide substitutions in vesicular stomatitis virus clearly showed that synonymous changes were roughly neutral [39]. The strong site-to-site variation in *dS* does suggest that selection is acting on synonymous substitutions in the HIV-1 *env* gene, but the highly similar distribution of rate variation implies that possible biases, if any, will affect all patients similarly**.** The similar site-to-site variation in *dS* for all patients also suggests that slower disease progression is not associated with stronger purifying selection, which could be a less likely alternative to explain the lower *dS* rates.

Because persistent immune activation is an important process underlying disease progression [19], it may provide a plausible explanation for differences in replication rates. The transcriptional machinery within the nucleus of the host cell is responsible for viral gene expression, making the HIV life cycle critically dependent on the activation state of the host cell [40]. HIV can infect resting CD4+ T cells, but after reverse transcription the pre-integration complex is degraded unless the cell is activated within a few days [41]. Immune activation, which is a strong predictor of progression to AIDS [20], is therefore a major stimulus for viral replication at the cellular level [40]. In addition, it is believed that T cell activation recruits large numbers of rapidly proliferating, short-lived, activated target CD4+ T cells, thereby creating the ideal condition for increased viral replication, while at the same time causing premature aging and exhaustion of the naïve T cell pool [23]. Experimental evidence indicates that this process is characterized by repeated bursts of viral replication, which have been suggested as a critical driver for the continuity of viral replication [19]. Transmission of free virus will only be efficient when target cells are in close proximity [e.g., 42], and infection of new cells is largely restricted to microscopic clusters of T cells in lymphoid tissue [e.g., 43], emphasizing the need for recruitment of uninfected activated CD4+ cells through immune activation. Because of both proliferation and activation of target cells, it is not surprising that the state of immune activation will affect HIV generation times and impose important constraints on viral evolution. The cause of immune activation is not clearly established, but it has been suggested that defective virus, which is the primary product of HIV replication, can drive immune activation [44]. If HIV itself plays a central role in immune activation, more replication will reinforce further the state of immune activation.

Recent findings show that Nef-mediated T cell receptor (TCR)–CD3 down-modulation is capable of protecting against immune activation and activation-induced cell death for most simian immunodeficiency viruses (SIV) [45]. This function has been generally conserved in primate lentiviral evolution but lost in the chimpanzee precursor of HIV-1, SIVcpz [45]. Therefore, these findings seem to resolve the longstanding issue of the nonpathogenic nature of SIV infection. In addition, it provides an explanation for the lower pathogenicity of HIV-2 infections [35] and the generally low HIV-2 synonymous substitution rates observed in this study. Nef-mediated TCR-CD3 down-modulation also correlated with CD4+ T cell depletion in SIVsmm infected sooty mangabeys [45]. A similar phenomenon might therefore be important for differences in pathogenicity in HIV-1 moderate and slow progressing patients. In contrast to an active suppression of immune activation in most SIV/HIV-2 infections, HIV-1 Nef can even increase immune activation [45], and patients infected with *nef*-defective HIV-1 have been reported to exhibit a slow progressing or even nonprogressing phenotype [46,47]. No doubt other viral and host factors will be important to explain differences between HIV/SIV infections. For example, SIVsmm infections usually exhibit high viral loads, while in asymptomatic HIV-2 infections viral loads are often too low to be detected. Recently, a low replicative capacity of HIV-2 compared with HIV-1 variants has been demonstrated [48]. Producing fewer viral particles per replication cycle will result in lower viral loads and may also constitute a lower stimulus for immune activation.

While immune activation provides a likely cause for the observed differences, there may be other explanations for a generation time effect in patients with different disease progression. For example, there may exist an evolutionary tradeoff between immune escape and replication rate. In the absence of immunological pressure, natural selection for fast replication rates would dominate viral evolution [49]. In the presence of an immune response, however, a tradeoff between immune escape and replication rate might be expected. There is evidence that the CTL escape response can indeed reduce replicative fitness by forcing the virus to endure detrimental CTL escape mutations [12,13]. Therefore, a vigorous CTL might be indirectly responsible for control of viral replication, longer HIV generation times, and slower disease progression. Differences in replication rate might also reflect differences in the contribution of the latent HIV reservoir to the circulating virus population. The pool of resting memory CD4+ T cells that carry integrated proviral genomes represents a stable reservoir for latent HIV infection. Although they may produce only a fraction of circulating viruses, modelling studies predict that such latently infected cells can still considerably impact mean generation times and replication rates [24,25]. The contribution of this reservoir to the free-floating virus population will become more important as CTL killing of infected activated CD4+ T cells becomes more efficient. Latently infected cells are also presumed to play a central role in the continuity of viral replication during chronic immune activation [19]. Between bursts of viral replication induced by immune activation, shedding of the virus in memory CD4+ T cells may be required [19]. Because it is hypothesized that replicative bursts are dependent on initial activation of these memory CD4+ T cells, this might be an additional mechanism by which viral generation times are affected by the state of immune activation. Multiple, overlapping, and nonsynchronized bursts will occur in spatially separated lymphoid tissue [19], which explains why viral replication may still be continuous and average genetic divergence can increase in an approximately linear fashion. Although CTL escape/viral attenuation and the dynamics of the latent reservoir can be involved in HIV generation times, they represent somewhat less direct explanations and require more experimental validation.

Our results show that the nonsynonymous rates in the *env* gene, most likely effecting phenotypic escape from nAbs (Figure 4, [2]), do not correlate with disease progression in patients harbouring HIV-1 group M viruses. While these conclusions are based on serial sampled data for only two patients, Frost et al. [2] have clearly demonstrated this effect in multiple patients. Crucially, we show that the selection pressure on *env* obscures the expected correlation between overall nucleotide substitution rates and disease progression, emphasizing the need to disentangle the contributions of synonymous and nonsynonymous substitutions to HIV evolution. Once these absolute rates are separated, we observe a significant decrease in *dN* rates after progression time, confirming the hypothesis of immune relaxation during the AIDS stage [32]. We conclude that this immune relaxation reflects attenuated humoral immunity, which is probably a consequence, rather than a cause, of variation in disease progression. This concept is supported by findings that AIDS patients with deteriorated immune responses usually have lower antibody titers than asymptomatic patients (e.g., [50]). Because it has been suggested that CXCR4-using viruses are subjected to stronger immune control in vivo [51], immune relaxation also provides an explanation of why these variants with a high in vitro replication capacity and increased target cell range generally do not appear until relatively late in HIV infection.

Our results suggest that nAbs are not an important factor in HIV disease progression. In this context, it is interesting to note that autologous nAb response is poor or nonexistent both in HIV-1 elite controllers [52] and HIV-2 infected patients (N. Taveira, unpublished data). However, previous studies have reported higher adaptation rates and more persistently positively selected sites in slow-progressing patients [17,18]. On the one hand, this discrepancy may suggest that nAbs, in particular broadly neutralizing antibodies, are able to moderate disease progression to some degree. On the other hand, we cannot exclude the possibility that adaptation rates and positive selected sites in *env* may reflect CTL selective pressure to some extent. Finally, phylodynamic processes, which explain the net viral adaptation rate as the interaction between viral abundance and selective pressure [53], must also be considered in HIV evolution. More research is needed to establish the relationship among immune responses, viral adaptation rates, and disease progression in HIV infection.

Although a procedure for estimating rates of synonymous and nonsynonymous substitution through time has been developed previously [22], there are some advantages to this approach. While Seo et al. [22] assume that the tree topology is known without error, we allow for uncertainty in the reconstructed genealogy. Our definition of internal and backbone branches is a phylogenetic one, but we average over a set of plausible trees in an empirical fashion and we also incorporate sampling time information. Seo et al. [22] were able to test whether changes in synonymous and nonsynonymous rates are correlated, whereas we model changes in the overall substitution rate (in an uncorrelated fashion [26]), after which the synonymous and nonsynonymous component for each branch is decoded using maximum likelihood methods. It is possible that a full probabilistic treatment of genealogical uncertainty and independent changes in synonymous and nonsynonymous rate could be developed. However, overcoming the technical hurdles required to achieve this objective is beyond the scope of this study. In this genealogical framework, we do not account for the process of within-host recombination, which can considerably shape HIV diversity [54]. Although recombination could lead to inflated *dN*/*dS* estimates [55], there is no reason to expect that recombination would affect synonymous rates in a way that correlates with disease progression. This was supported by the lack of clear correlations between *dS* and recombination rate estimates.

In conclusion, our evolutionary analysis revealed a strong correlation between synonymous substitution rates and HIV disease progression. Further work will be necessary, however, to clearly establish the mechanisms that determine HIV generation times. A combined approach incorporating both experimental and computational techniques should provide important insights into the patterns of HIV evolution and disease progression.

## Materials and Methods

### Estimating absolute rates of synonymous and nonsynonymous substitution.

Our approach to infer synonymous and nonsynonymous substitution rates, and to explore how these rates change through time, is an empirical extension of recently developed Bayesian relaxed-clock models [26]. Given a fixed tree topology, branch lengths measured in units of expected synonymous and nonsynonymous substitutions can be estimated using codon substitution models. We applied a codon-based extension of the Hasegawa–Kishino–Yano model (HKY85) (MG94xHKY85 with codon equilibrium frequencies estimated from position-specific nucleotide frequencies, [56]), for which the rate matrix for substituting codon *x* with codon *y* in infinitesimal time, *Q_x,y_* (α,β,κ), is given by:

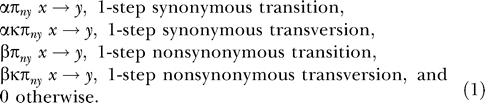
In this parameterization, α denotes the synonymous substitution rate, while β denotes the nonsynonymous substitution rate, and their ratio (ω = β/α) reflects the strength of selective pressure along a specific branch. κ is the transversion/transition ratio, and π*_ny_* represents the frequency of the target nucleotide at the appropriate codon position. The parameters α and β can be shared among branches (equivalent to the “one-ratio” model; [57]) or allowed to take up branch-specific values (equivalent to the “free-ratio” model; [57]). We applied the latter, dubbed the “local” codon model, which has the following form for the expected number of substitutions per site on branch *b_i_*:

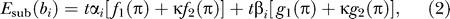
where *f*_1_, *f*_2_, *g*_1_, and *g*_2_ are functions determined by the nucleotide composition of the sequence alignment and shared among branches [58]. The first term in the sum corresponds to the contribution of synonymous substitutions (a product of the branch-specific α) and the second to the contribution of nonsynonymous substitutions (a product of the branch-specific β). The time parameter *t* is not estimable alone but products *t*α*_i_* and *t*β*_i_* are.

In this codon model, the time parameter *t* could be estimated assuming a dated tip molecular clock model, thereby providing absolute rates of synonymous and nonsynonymous substitution [59]. To estimate changes in synonymous and nonsynonymous rates, however, the assumption of a strict molecular clock needs to be relaxed (e.g., [22]). Recently, a relaxed-clock approach has been developed that is applicable to measurably evolving populations and takes into account genealogical uncertainty by averaging over a set of plausible trees [26]. Since the sampling of genealogies using codon models is too computationally expensive at present, we applied an empirical extension of the relaxed phylogenetic model and sample trees under the equivalent nucleotide substitution model.

We used Markov chain Monte Carlo (MCMC) methods as implemented in BEAST 1.3 to obtain a posterior distribution of trees under an uncorrelated relaxed clock [60,61]. In this approach, the nucleotide substitution rate on each branch of the tree is drawn independently and identically from an underlying rate distribution [26], in this case an exponential prior distribution among branches. Our full Bayesian probabilistic treatment is characterized by the following posterior distribution:

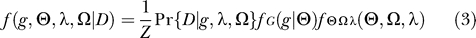
where *g* represents the tree topology and Θ contains the hyperparameters of the tree prior. We used a piecewise-constant model of population size, the Bayesian skyline plot model, that provides population size estimates for each coalescent interval of the genealogy *g* [62]. Θ contains both the parameters for the group sizes, which define the number of coalescent events in each grouped interval, and the population size for each group. The relaxed-clock parameter λ represents the exponential distribution of rates across lineages (with mean and standard deviation λ^−1^) [26]. Because we used the HKY85 of nucleotide substitution with gamma-distributed rate variation among sites, the vector Ω contains the transition/transversion ratio (κ) and the shape of the gamma distribution (α). The posterior density was investigated using MCMC with the length of the chains, sampling frequency, and burn-in dependent on the dataset analyzed. The MCMC samples were inspected for convergence to stationarity, and effective sampling sizes were calculated using Tracer 1.2 [63].

Using the relaxed-clock approach, a posterior distribution of tree topologies can be obtained with branch lengths in units of time and in units of the expected number of nucleotide substitution per site, both related by the vector of rates for the branches [26]. To infer branch lengths in the expected number of synonymous and nonsynonymous substitutions separately, we applied the local codon model to a set of trees sampled from the posterior distribution. To achieve computational tractability and convergence under this parameter-rich scheme (for reasonably large datasets, both in terms of the number of taxa and the number of posterior trees), we inferred the maximum likelihood estimates of the parameters while constraining the branch lengths (*E*_sub_) to the codon-rescaled estimates obtained by the Bayesian relaxed-clock analysis. This allowed us to determine the nonsynonymous and synonymous component of the branch lengths, and using the Bayesian estimate for branches in time units for each tree, absolute rates of nonsynonymous and synonymous substitutions could be inferred and empirically averaged over a set of genealogies. It has been shown that fixing branch lengths has a negligible effect on secondary nonsynonymous and synonymous rate inference [64]. For each dataset, maximum likelihood estimation was performed using HYPHY version 0.99 based on 200 trees sampled from the posterior distribution [65]. Scripts to perform local codon analysis on a set of trees in HYPHY are available at http://evolve.zoo.ox.ac.uk and http://www.hyphy.org. Within-gene variation of synonymous and nonsynonymous substitution rates was analyzed using codon models that incorporate site-to-site heterogeneity of both *dS* and *dN* [30].

Under the uncorrelated relaxed clock, it is possible to infer individual rates for all branches in a genealogy and, thus, also a mean rate for each subset of branches. Our analysis focuses on within-host HIV genealogies, for which terminal branches may have an excess of short-lived deleterious mutations that have not reached fixation. Therefore, we estimated mean substitution rates for internal branches and for the central trunk of the ladder-like genealogies, referred to as the “backbone” (Figure 1). We define the backbone as the set of branches connecting the root of the tree to the sequences sampled at the last time point, excluding both terminal branches and internal branches from a common ancestor of sequences sampled at the last time point only. Weighted averages were used to report mean substitution rates for a set of branches, as defined by Drummond et al. [26]. Mean substitution rates before or after progression time were calculated as weighted averages for the set of branches (or partial branches) before or after that time point in the tree. Nonsynonymous and synonymous divergence over time was estimated by calculating the mean accumulation of substitutions along the lineages in a particular time interval and averaging them over the set of genealogies. Software to plot divergence over time and to obtain substitution rates for a subset of branches is available from the authors on request.

### Datasets.

Substitution rates in relationship to disease progression were investigated in nine patients extensively sampled over a 6–13.7 year period starting close to the time of seroconversion [14,27]. Both the Shankarappa et al. [14] C2V3 *env* sequence data and the Shriner et al. (2004) [27] follow-up sequences were analyzed. Complete *env* gene sequences longitudinally sampled from two patients with distinct phenotypic escape form nAbs were obtained from Frost et al. [2]. Heterochronous HIV-1 group O *env* data were obtained from the two available intrapatient evolution studies [33,36]. For HIV-2, we analyzed *env* clones from two serially sampled patients [66]; the sequence “C9/1997” from patient C was excluded from the analysis because it has been identified previously as a recombinant [67]. In addition, we inferred substitution rates from population sequences sampled at different time points in four separate patients [68]. Since only three or four sequences were available per patient, these sequences were analyzed as one single dataset with the internal and backbone rate calculated as the mean rate for only within-host internal branches and backbone branches, respectively. Finally, we analyzed data from three HIV-2 mother–child transmission pairs [37], including unpublished sequences for one pair (P3P4). These sequences were obtained using methods described elsewhere [37] and are available from GenBank. Since only one sample of cloned sequences was available for both mother and child at the same time, rates were estimated by specifying a normal prior distribution on the time to the most recent common ancestor based on the time of birth; monophyletic constraints were imposed on both the mother and child sequences. HIV-1 group M and HIV-2 alignments were trimmed so that approximately the same gene region was investigated for all datasets (position 823 to 1128 relative to HXB2 *env* and position 823 to 1134 relative to SMM239 *env*).

## Supporting Information

Figure S1 Genealogies for Patients 2 (A), 3 (B), 5 (C), 6 (D), 7 (E), 8 (F), 9 (G), and 11 (H) Indicating Backbone, Internal, and External Branches The trees for each patient were selected from the Bayesian relaxed-clock posterior distribution using a maximum clade credibility criterion. Backbone, internal, and external branches are shown in red, blue, and black, respectively.
(684 KB PDF)

Figure S2 Scatter Plots of Mean Synonymous (A) and Nonsynonymous (B) Substitution Rates on Internal Branches as a Function of Progression Time, CD4+ T Cell Count Slope, and Log Viral Load Slope Rates as a function of progression time and the slope of the CD4+ T cell count are shown in pink, while rates as a function of the log *VL* slope are shown in cyan. The slope of the CD4+ T cell count and log *VL* were calculated based on linear regression of these parameters as a function of time. The error bars represent the standard errors of the estimates. There is a significant correlation between the log of synonymous rates on internal branches and progression (*r* = −0.74, *p* = 0.023). The same is true for the correlation with CD4+ T cell count change over time (*r* = −0.73, *p* = 0.026). There is no significant correlation with log *VL* change over time (*r* = 0.53, *p* = 0.142). No correlations are significant for nonsynonymous rates.
Found at DOI: 10.1371/journal.pcbi.0030029.sg002 (875 KB AI).

Figure S3 Scatter Plots of Mean Synonymous Substitution Rates on Backbone (A) and Internal (B) Branches for a Restricted Number of Time Points as a Function of Progression Time, CD4+ T Cell Count Slope, and Log Viral Load Slope Rates were inferred for datasets restricted to time points up to about 70 months after seroconversion (last included time point at 77, 73, 73, 68, 73, 74, 70, 63, and 70 months for patients 1, 2, 3, 5, 6, 7, 8, 9, and 11, respectively). Rates as a function of progression time and the slope of the CD4+ T cell count are shown in pink, while rates as a function of the log *VL* slope are shown in cyan. The slope of the CD4+ T cell count and log *VL* were calculated based on linear regression of these parameters as a function of time. The error bars represent the standard errors of the estimates. The log of the backbone *dS* rate shows a moderate negative correlation with progression time (*r* = −0.64, *p* = 0.066) and a moderate positive correlation with the change in log *VL* (*r* = 0.58, *p* = 0.10). There is a stronger correlation with the rate of CD4+ T cell count change over time (*r* = −0.73, *p* = 0.023). The log of internal rates is also significantly correlated with progression time (*r* = −0.68, *p* = 0.043) and the rate of CD4+ T cell count change over time (*r* = −0.74, *p* = 0.022).
Found at DOI: 10.1371/journal.pcbi.0030029.sg003 (831 KB AI).

Table S1 Mean Internal/External Rate Ratios for Simulated and Real Datasets For each patient, five genealogies were simulated with the same amount of serial samples and sequences per sample under both an exponential growth (Expo) and a logistic growth (Log) model. Branch lengths in time units were multiplied with the mean evolutionary rate and rescaled to codon substitutions per codon site. Finally, sequences were evolved along the trees using a “single *dN*/*dS*” codon model [69]. We also applied the same procedure but allowed rate variation by rescaling branch lengths using branch-specific rates, drawn identically and independently from an exponential distribution. Both for genealogy and sequence simulation, the parameters inferred from the real data were used. The internal/external rate ratios for each patient are listed at the bottom of the table (Real). For seven patients, the real internal/external rate ratio is smaller than all the simulated datasets. For two patients (3 and 9), who have the highest internal/external rate ratio, only a single simulated dataset has a ratio lower than the real value. The *p*-value is the probability of obtaining the real internal/external rate ratio by chance alone assuming neutrality and assuming that the 20 simulated datasets are equivalent replicates of the within-host neutral evolutionary process.
(99 KB DOC)

Table S2 Recombination Rate Estimates for Each Time Point Recombination rates were estimated using the composite likelihood estimator [70], which has been extended to accommodate for the general time-reversible model of nucleotide substitution [71]. Recombination rates (rho) are listed for each available time point *(Tp)*. The symbol * indicates that the likelihood permutation test was significant (*p* < 0.05). For each sample, the time since seroconversion *(Ts)* in months and the number of segregating sites *(S)* in the sequence sample is shown. The standard deviations *(SD)* were obtained by repeating the estimation process ten times.
(172 KB DOC)

Table S3 Analysis of Among-Site Nonsynonymous and Synonymous Rate Variation For each patient, log likelihoods are listed for a model that allows nonsynonymous rate variation (NS) and for a model that allows both nonsynonymous and synonymous rate variation (Dual) among sites [30]. Reported *p*-value is for the likelihood ratio test between the NS and Dual rate variation models, using the χ^2^ distribution with four degrees of freedom. Coefficients of variation (CV) are listed for *dN* and *dS* in the Dual model.
(36 KB DOC)

Table S4 Mean Synonymous and Nonsynonymous Substitution Rates for Different HIV Variants Substitution rates and standard deviations are listed in substitutions per site per month for HIV-1 group M (subtype B, 14), HIV-1 group O [33,36], and HIV-2 [37,63,65] datasets. The HIV-1 group M infected patients are ordered according to increasing progression time. P3P4, P7P8, and P9P10 are HIV-2 transmission chains (see Methods); the estimates therefore represent the mean evolutionary rate in two infected patients. The estimates for Shi et al. [68] represent mean substitution rates in four individuals for which only limited sequences were available (see Methods).
(60 KB DOC)

### Accession numbers

The GenBank (http://www.ncbi.nlm.nih.gov/GenBank) accession numbers of the HIV-2 sequences discussed in this paper are DQ787116–DQ787121.

## Abbreviations

CTL: cytotoxic T cell
*dN*: nonsynonymous
*dS*: synonymous
HKY85: Hasegawa–Kishino–Yano model
HLA: human leukocyte antigen
log *VL*: log viral load
MCMC: Markov chain Monte Carlo
nAb: neutralizing antibody
SIV: simian immunodeficiency virus
TCR: T cell receptor

## References

[pcbi-0030029-b001] Richman DD, Wrin T, Little SJ, Petropoulos CJ (2003). Rapid evolution of the neutralizing antibody response to HIV type 1 infection. Proc Natl Acad Sci U S A.

[pcbi-0030029-b002] Frost SD, Wrin T, Smith DM, Kosakovsky Pond SL, Liu Y (2005). Neutralizing antibody responses drive the evolution of human immunodeficiency virus type 1 envelope during recent HIV infection. Proc Natl Acad Sci U S A.

[pcbi-0030029-b003] Cecilia D, Kleeberger C, Munoz A, Giorgi JV, Zolla-Pazner S (1999). A longitudinal study of neutralizing antibodies and disease progression in HIV-1-infected subjects. J Infect Dis.

[pcbi-0030029-b004] Koup RA, Safrit JT, Cao Y, Andrews CA, McLeod G (1994). Temporal association of cellular immune responses with the initial control of viremia in primary human immunodeficiency virus type 1 syndrome. J Virol.

[pcbi-0030029-b005] Carrington M, Nelson GW, Martin MP, Kissner T, Vlahov D (1999). HLA and HIV-1: Heterozygote advantage and B*35-Cw*04 disadvantage. Science.

[pcbi-0030029-b006] Trachtenberg E, Korber B, Sollars C, Kepler TB, Hraber PT (2003). Advantage of rare HLA supertype in HIV disease progression. Nat Med.

[pcbi-0030029-b007] Asquith B, Edwards CT, Lipsitch M, McLean AR (2006). Inefficient cytotoxic T lymphocyte-mediated killing of HIV-1-infected cells in vivo. PLoS Biol.

[pcbi-0030029-b008] Addo MM, Yu XG, Rathod A, Cohen D, Eldridge RL (2003). Comprehensive epitope analysis of human immunodeficiency virus type 1 (HIV-1)-specific T-cell responses directed against the entire expressed HIV-1 genome demonstrate broadly directed responses, but no correlation to viral load. J Virol.

[pcbi-0030029-b009] Cao J, McNevin J, Holte S, Fink L, Corey L (2003). Comprehensive analysis of human immunodeficiency virus type 1 (HIV-1)-specific gamma interferon-secreting CD8+ T cells in primary HIV-1 infection. J Virol.

[pcbi-0030029-b010] Lichterfeld M, Yu XG, Cohen D, Addo MM, Malenfant J (2004). HIV-1 *nef* is preferentially recognized by CD8 T cells in primary HIV-1 infection despite a relatively high degree of genetic diversity. AIDS.

[pcbi-0030029-b011] Goulder PJ, Watkins DI (2004). HIV and SIV CTL escape: Implications for vaccine design. Nat Rev Immunol.

[pcbi-0030029-b012] Martinez-Picado J, Prado JG, Fry EE, Pfafferott K, Leslie A (2006). Fitness cost of escape mutations in p24 *gag* in association with control of human immunodeficiency virus type 1. J Virol.

[pcbi-0030029-b013] Leslie AJ, Pfafferott KJ, Chetty P, Draenert R, Addo MM (2004). HIV evolution: CTL escape mutation and reversion after transmission. Nat Med.

[pcbi-0030029-b014] Shankarappa R, Margolick JB, Gange SJ, Rodrigo AG, Upchurch D (1999). Consistent viral evolutionary changes associated with the progression of human immunodeficiency virus type 1 infection. J Virol.

[pcbi-0030029-b015] Ganeshan S, Dickover RE, Korber BT, Bryson YJ, Wolinsky SM (1997). Human immunodeficiency virus type 1 genetic evolution in children with different rates of development of disease. J Virol.

[pcbi-0030029-b016] Strunnikova N, Ray SC, Livingston RA, Rubalcaba E, Viscidi RP (1995). Convergent evolution within the V3 loop domain of human immunodeficiency virus type 1 in association with disease progression. J Virol.

[pcbi-0030029-b017] Williamson S (2003). Adaptation in the *env* gene of HIV-1 and evolutionary theories of disease progression. Mol Biol Evol.

[pcbi-0030029-b018] Ross HA, Rodrigo AG (2002). Immune-mediated positive selection drives human immunodeficiency virus type 1 molecular variation and predicts disease duration. J Virol.

[pcbi-0030029-b019] Grossman Z, Meier-Schellersheim M, Paul WE, Picker LJ (2006). Pathogenesis of HIV infection: What the virus spares is as important as what it destroys. Nat Med.

[pcbi-0030029-b020] Giorgi JV, Hultin LE, McKeating JA, Johnson TD, Owens B (1999). Shorter survival in advanced human immunodeficiency virus type 1 infection is more closely associated with T lymphocyte activation than with plasma virus burden or virus chemokine coreceptor usage. J Infect Dis.

[pcbi-0030029-b021] Sousa AE, Carneiro J, Meier-Schellersheim M, Grossman Z, Victorino RM (2002). CD4 T cell depletion is linked directly to immune activation in the pathogenesis of HIV-1 and HIV-2 but only indirectly to the viral load. J Immunol.

[pcbi-0030029-b022] Seo TK, Kishino H, Thorne JL (2004). Estimating absolute rates of synonymous and nonsynonymous nucleotide substitution in order to characterize natural selection and date species divergences. Mol Biol Evol.

[pcbi-0030029-b023] Silvestri G, Feinberg MB (2003). Turnover of lymphocytes and conceptual paradigms in HIV infection. J Clin Invest.

[pcbi-0030029-b024] Kelly JK (1996). Replication rate and evolution in the human immunodeficiency virus. J Theor Biol.

[pcbi-0030029-b025] Kelly JK, Williamson S, Orive ME, Smith MS, Holt RD (2003). Linking dynamical and population genetic models of persistent viral infection. Am Nat.

[pcbi-0030029-b026] Drummond AJ, Ho SYW, Phillips MJ, Rambaut A (2006). Relaxed phylogenetics and dating with confidence. PLoS Biol.

[pcbi-0030029-b027] Shriner D, Shankarappa R, Jensen MA, Nickle DC, Mittler JE (2004). Influence of random genetic drift on human immunodeficiency virus type 1 *env* evolution during chronic infection. Genetics.

[pcbi-0030029-b028] Edwards CTT, Holmes EC, Pybus OG, Wilson DJ, Viscidi RP (2006). Evolution of the human immunodeficiency virus envelope gene is dominated by negative selection. Genetics.

[pcbi-0030029-b029] Kosakovsky Pond SL, Frost SD, Grossman Z, Gravenor M, Richman DD (2006). Adaptation to different human populations by HIV-1 revealed by codon-based analysis. PloS Comput Biol.

[pcbi-0030029-b030] Kosakovsky Pond SL, Muse SV (2005). Site-to-site variation of synonymous substitution rates. Mol Biol Evol.

[pcbi-0030029-b031] Frost SD, Dumaurier MJ, Wain-Hobson S, Brown AJ (2001). Genetic drift and within-host metapopulation dynamics of HIV-1 infection. Proc Natl Acad Sci U S A.

[pcbi-0030029-b032] Williamson S, Perry SM, Bustamante CD, Orive ME, Stearns MN (2005). A statistical characterization of consistent patterns of human immunodeficiency virus evolution within infected patients. Mol Biol Evol.

[pcbi-0030029-b033] Janssens W, Nkengasong J, Heyndrickx L, Van der Auwera G, Vereecken K (1999). Intrapatient variability of HIV type 1 group O ANT70 during a 10-year follow-up. AIDS Res Hum Retroviruses.

[pcbi-0030029-b034] O'Donovan D, Ariyoshi K, Milligan P, Ota M, Yamuah L (2000). Maternal plasma viral RNA levels determine marked differences in mother-to-child transmission rates of HIV-1 and HIV-2 in The Gambia. MRC/Gambia Government/University College London Medical School working group on mother-child transmission of HIV. AIDS.

[pcbi-0030029-b035] Marlink R, Kanki P, Thior I, Travers K, Eisen G (1994). Reduced rate of disease development after HIV-2 infection as compared to HIV-1. Science.

[pcbi-0030029-b036] Chaix-Baudier ML, Chappey C, Burgard M, Letourneur F, Igual J (1998). First case of mother-to-infant HIV type 1 group O transmission and evolution of C2V3 sequences in the infected child. French HIV Pediatric Cohort Study Group. AIDS Res Hum Retroviruses.

[pcbi-0030029-b037] Barroso H, Araujo F, Gomes MH, Mota-Miranda A, Taveira N (2004). Phylogenetic demonstration of two cases of perinatal human immunodeficiency virus type 2 infection diagnosed in adulthood. AIDS Res Hum Retroviruses.

[pcbi-0030029-b038] Choisy M, Woelk CH, Guegan JF, Robertson DL (2004). Comparative study of adaptive molecular evolution in different human immunodeficiency virus groups and subtypes. J Virol.

[pcbi-0030029-b039] Sanjuan R, Moya A, Elena SF (2004). The distribution of fitness effects caused by single-nucleotide substitutions in an RNA virus. Proc Natl Acad Sci U S A.

[pcbi-0030029-b040] Lawn SD, Butera ST, Folks TM (2001). Contribution of immune activation to the pathogenesis and transmission of human immunodeficiency virus type 1 infection. Clin Microbiol Rev.

[pcbi-0030029-b041] Bukrinsky MI, Stanwick TL, Dempsey MP, Stevenson M (1991). Quiescent T lymphocytes as an inducible virus reservoir in HIV-1 infection. Science.

[pcbi-0030029-b042] Dimitrov DS, Willey RL, Sato H, Chang LJ, Blumenthal R (1993). Quantitation of human immunodeficiency virus type 1 infection kinetics. J Virol.

[pcbi-0030029-b043] Hosmalin A, Samri A, Dumaurier MJ, Dudoit Y, Oksenhendler E (2001). HIV-specific effector cytotoxic T lymphocytes and HIV-producing cells colocalize in white pulps and germinal centers from infected patients. Blood.

[pcbi-0030029-b044] Finzi D, Plaeger SF, Dieffenbach CW (2006). Defective virus drives human immunodeficiency virus infection, persistence, and pathogenesis. Clin Vaccine Immunol.

[pcbi-0030029-b045] Schindler M, Munch J, Kutsch O, Li H, Santiago ML (2006). *Nef*-mediated suppression of T cell activation was lost in a lentiviral lineage that gave rise to HIV-1. Cell.

[pcbi-0030029-b046] Deacon NJ, Tsykin A, Solomon A, Smith K, Ludford-Menting M (1995). Genomic structure of an attenuated quasi species of HIV-1 from a blood transfusion donor and recipients. Science.

[pcbi-0030029-b047] Kirchhoff F, Greenough TC, Brettler DB, Sullivan JL, Desrosiers RC (1995). Brief report: Absence of intact *nef* sequences in a long-term survivor with nonprogressive HIV-1 infection. N Engl J Med.

[pcbi-0030029-b048] Blaak H, van der Ende ME, Boers PH, Schuitemaker H, Osterhaus AD (2006). In vitro replication capacity of HIV-2 variants from long-term aviremic individuals. Virology.

[pcbi-0030029-b049] Nowak M, May RM (2000). Virus dynamics.

[pcbi-0030029-b050] Ljunggren K, Moschese V, Broliden PA, Giaquinto C, Quinti I (1990). Antibodies mediating cellular cytotoxicity and neutralization correlate with a better clinical stage in children born to human immunodeficiency virus-infected mothers. J Infect Dis.

[pcbi-0030029-b051] Bonhoeffer S, Holmes EC, Nowak MA (1995). Causes of HIV diversity. Nature.

[pcbi-0030029-b052] Bailey JR, Lassen KG, Yang HC, Quinn TC, Ray SC (2006). Neutralizing antibodies do not mediate suppression of human immunodeficiency virus type 1 in elite suppressors or selection of plasma virus variants in patients on highly active antiretroviral therapy. J Virol.

[pcbi-0030029-b053] Grenfell BT, Pybus OG, Gog JR, Wood JL, Daly JM (2004). Unifying the epidemiological and evolutionary dynamics of pathogens. Science.

[pcbi-0030029-b054] Shriner D, Rodrigo AG, Nickle DC, Mullins JI (2004). Pervasive genomic recombination of HIV-1 in vivo. Genetics.

[pcbi-0030029-b055] Anisimova M, Nielsen R, Yang Z (2003). Effect of recombination on the accuracy of the likelihood method for detecting positive selection at amino acid sites. Genetics.

[pcbi-0030029-b056] Kosakovsky Pond SL, Frost SD (2005). A simple hierarchical approach to modeling distributions of substitution rates. Mol Biol Evol.

[pcbi-0030029-b057] Yang Z (1998). Likelihood ratio tests for detecting positive selection and application to primate lysozyme evolution. Mol Biol Evol.

[pcbi-0030029-b058] Kosakovsky Pond SL, Muse SV, Nielsen R (2004). HyPhy: Hypothesis testing using phylogenies. Statistical methods in molecular evolution.

[pcbi-0030029-b059] Rambaut A (2000). Estimating the rate of molecular evolution: Incorporating non-contemporaneous sequences into maximum likelihood phylogenies. Bioinformatics.

[pcbi-0030029-b060] Drummond AJ, Nicholls GK, Rodrigo AG, Solomon W (2002). Estimating mutation parameters, population history and genealogy simultaneously from temporally spaced sequence data. Genetics.

[pcbi-0030029-b061] Drummond A, Rambaut A (2003). BEAST version 1.3 [computer program].

[pcbi-0030029-b062] Drummond AJ, Rambaut A, Shapiro B, Pybus OG (2005). Bayesian coalescent inference of past population dynamics from molecular sequences. Mol Biol Evol.

[pcbi-0030029-b063] Rambaut A, Drummond AJ (2003). Tracer, version 1.2 [computer program].

[pcbi-0030029-b064] Kosakovsky Pond SL, Frost SD (2005). Not so different after all: A comparison of methods for detecting amino-acid sites under selection. Mol Biol Evol.

[pcbi-0030029-b065] Kosakovsky Pond SL, Frost SD, Muse SV (2005). HyPhy: Hypothesis testing using phylogenies. Bioinformatics.

[pcbi-0030029-b066] Esteves A, Piedade J, Santos C, Venenno T, Canas-Ferreira WF (2001). Follow-up study of intrahost HIV type 2 variability reveals discontinuous evolution of C2V3 sequences. AIDS Res Hum Retroviruses.

[pcbi-0030029-b067] Gottlieb GS, Mullins JI (2001). Re: Follow-up study of intrahost HIV type 2 variability reveals discontinuous evolution of C2V3 sequences. AIDS Res Hum Retroviruses.

[pcbi-0030029-b068] Shi Y, Brandin E, Vincic E, Jansson M, Blaxhult A (2005). Evolution of human immunodeficiency virus type 2 coreceptor usage, autologous neutralization, envelope sequence and glycosylation. J Gen Virol.

[pcbi-0030029-b069] Nielsen R, Yang Z (1998). Likelihood models for detecting positively selected amino acid sites and applications to the HIV-1 envelope gene. Genetics.

[pcbi-0030029-b070] McVean G, Awadalla P, Fearnhead P (2002). A coalescent-based method for detecting and estimating recombination from gene sequences. Genetics.

[pcbi-0030029-b071] Carvajal-Rodriguez A, Crandall KA, Posada D (2006). Recombination estimation under complex evolutionary models with the coalescent composite-likelihood method. Mol Biol Evol.

