# Supplementary material for: Synonymous Substitution Rates Predict HIV Disease Progression as a Result of Underlying Replication Dynamics
Source: PLoS Comput Biol. 2007 Feb 23;3(2):e29. doi: 10.1371/journal.pcbi.0030029 (PMC1797821; doi:10.1371/journal.pcbi.0030029)

Figure S1

A)

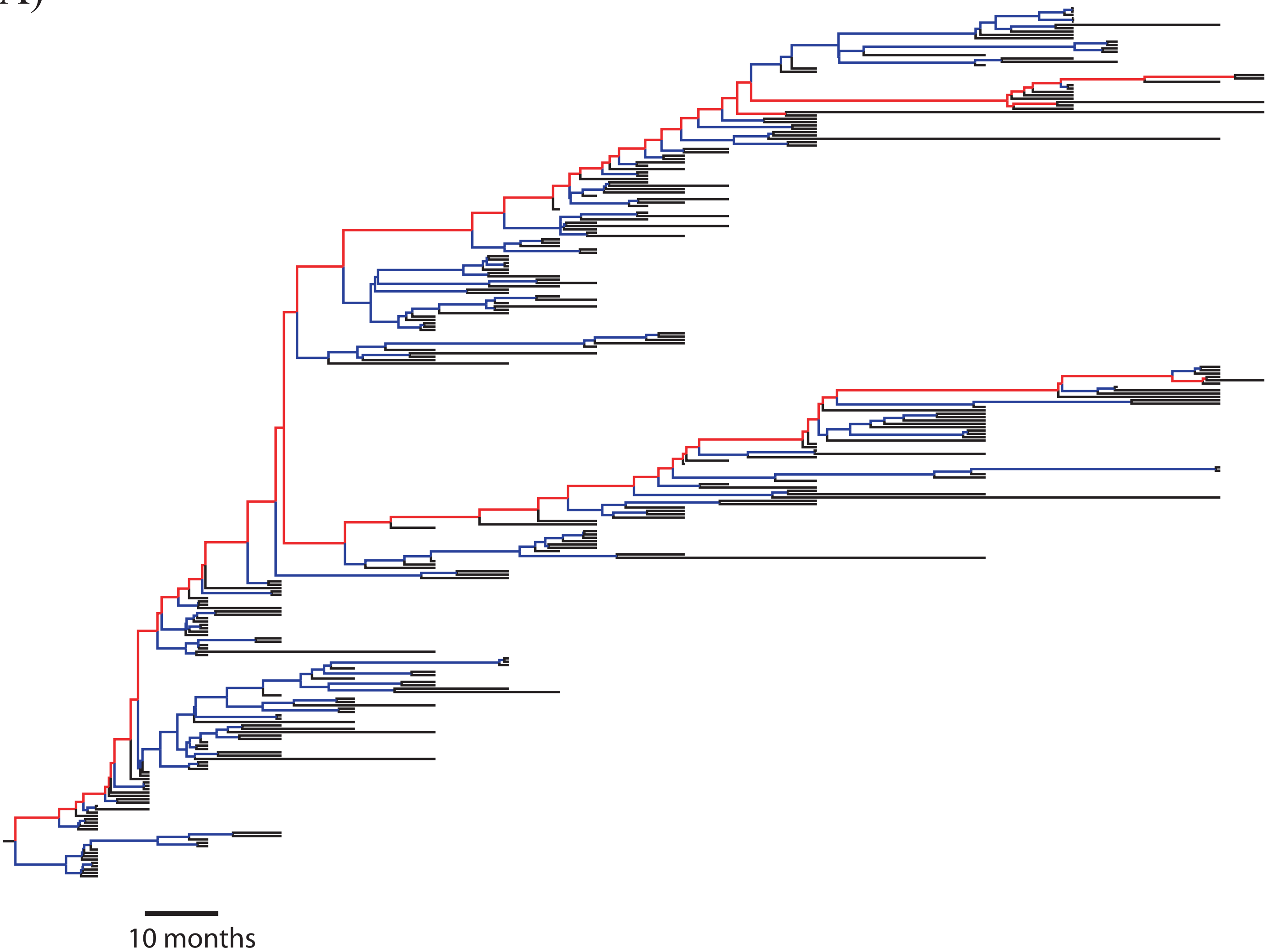

B)

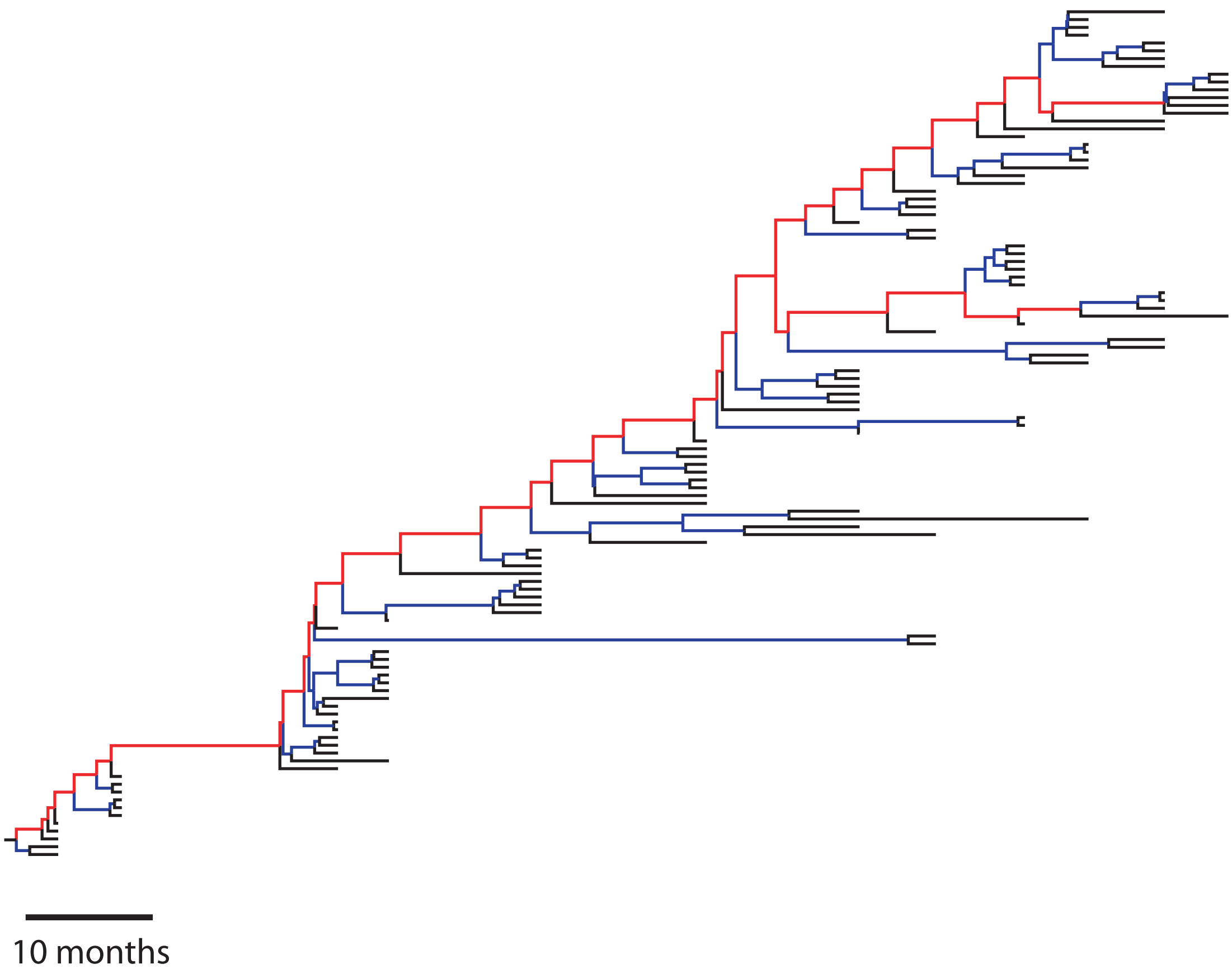

Figure S1

C)

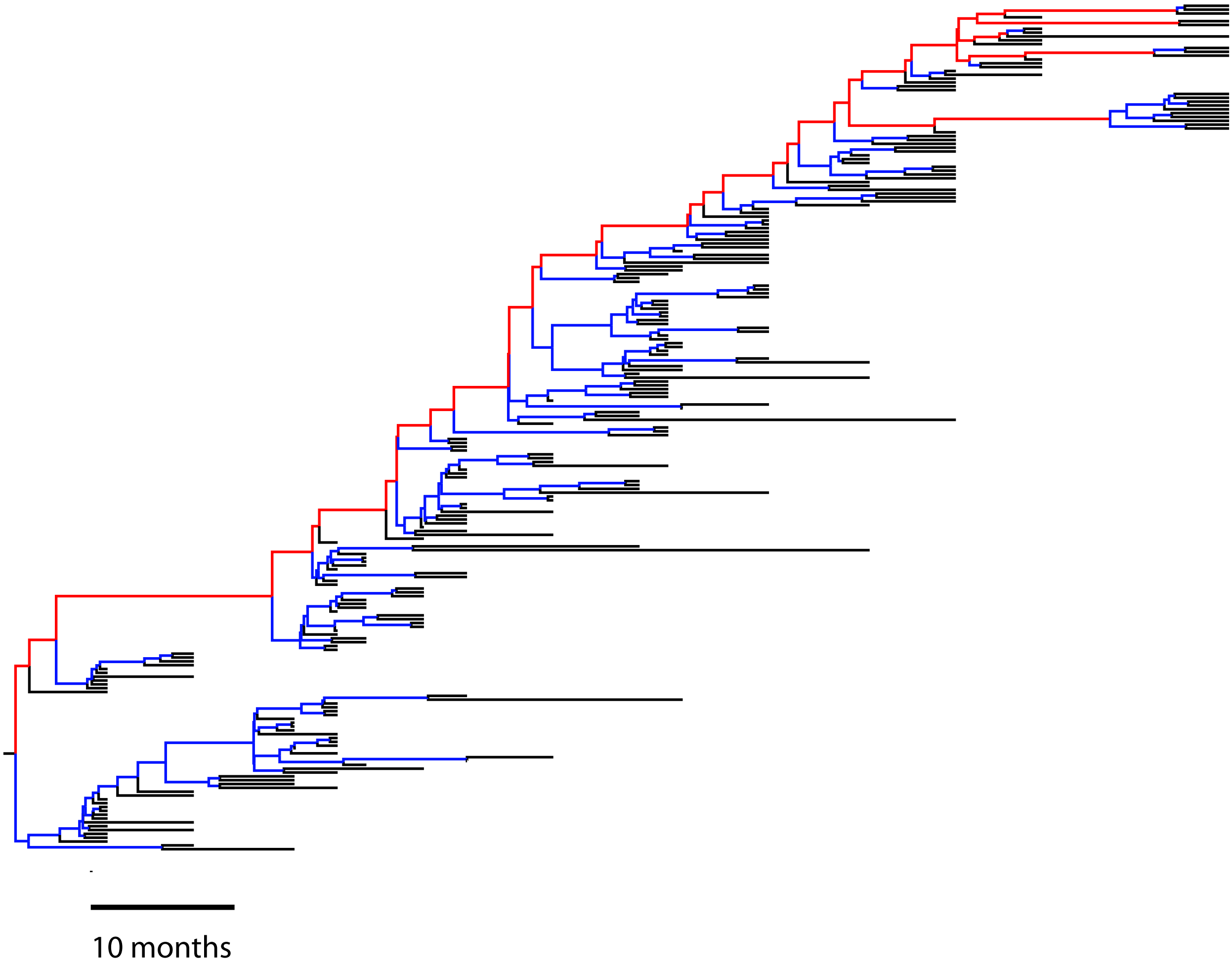

D)

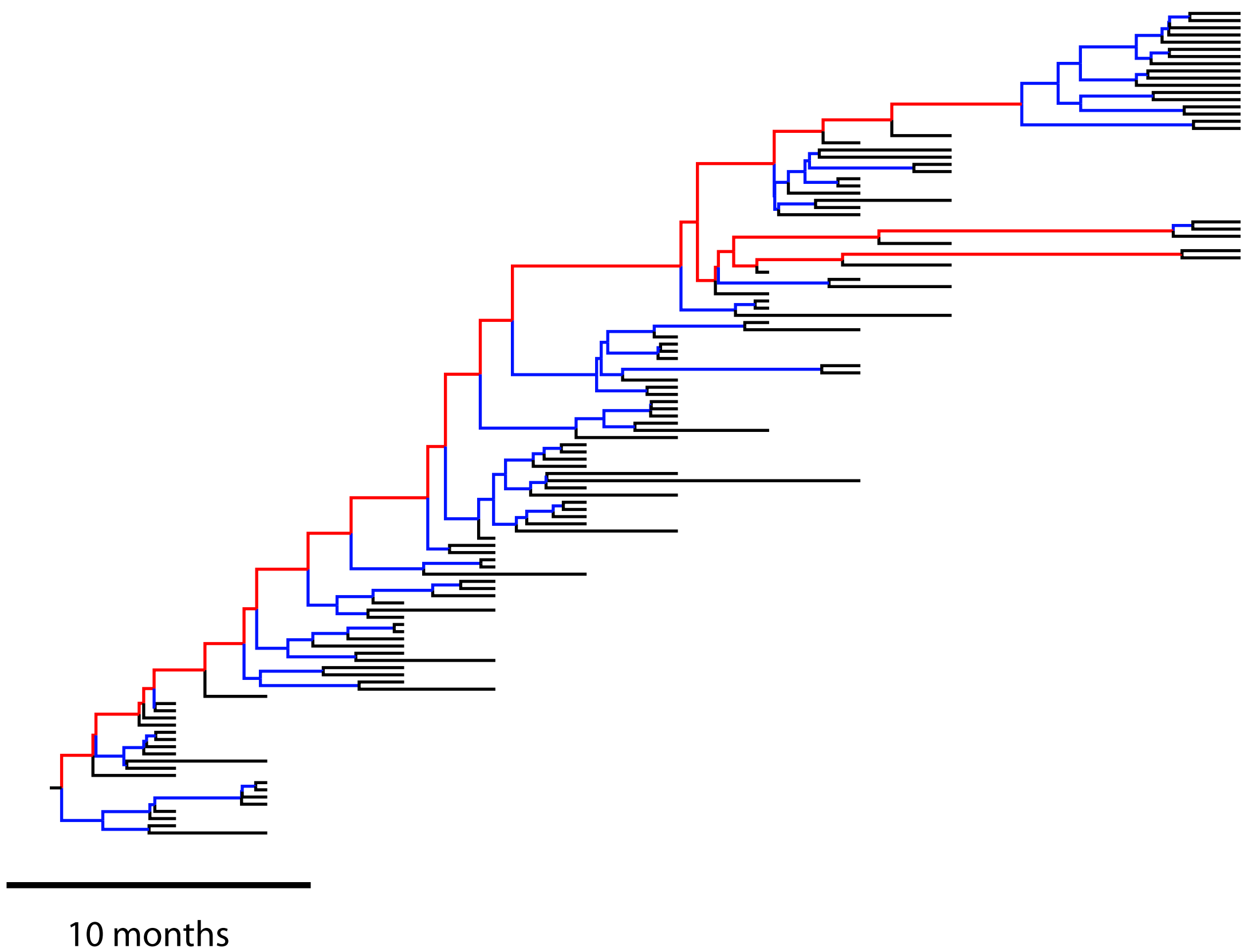

Figure S1

E)

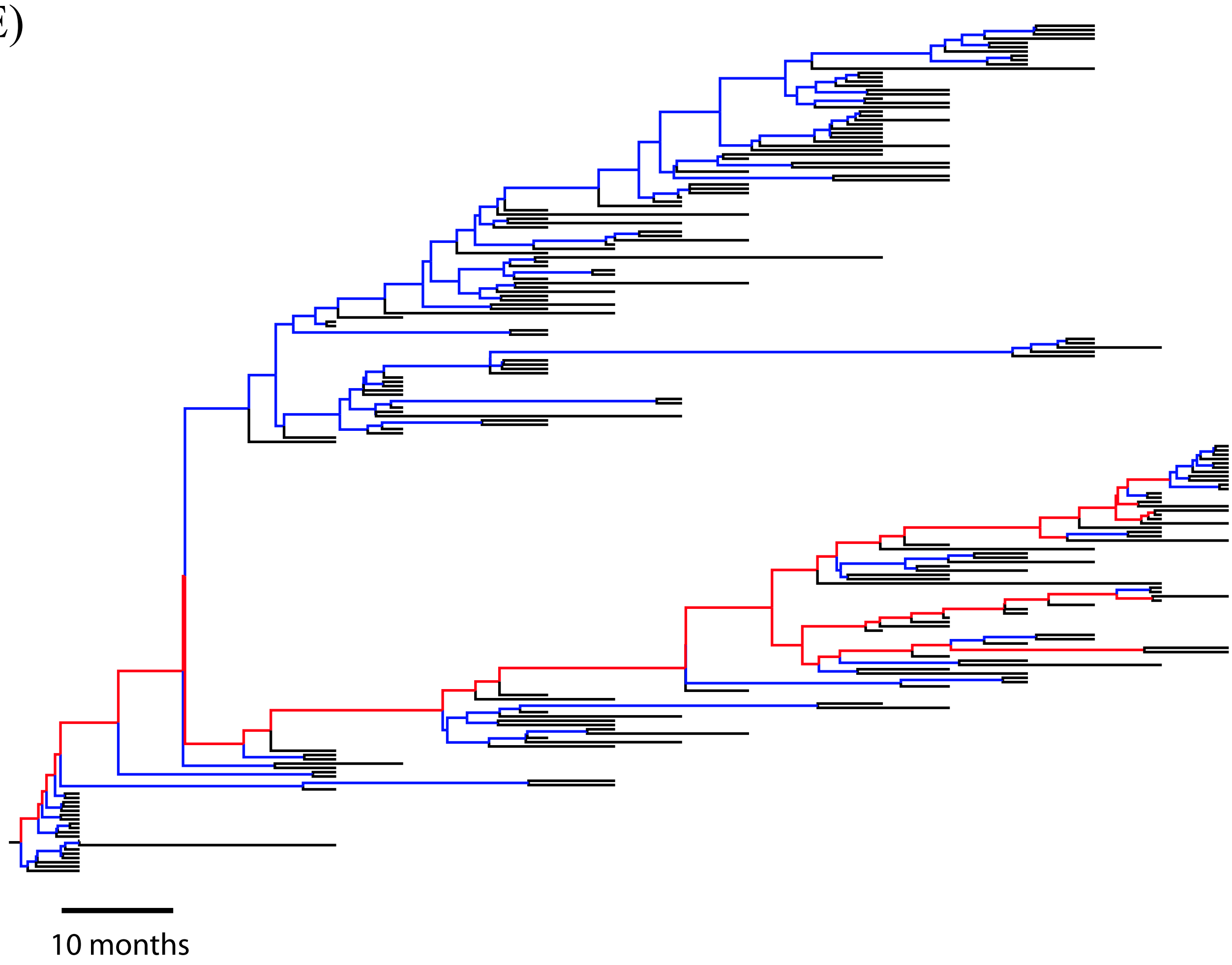

F)

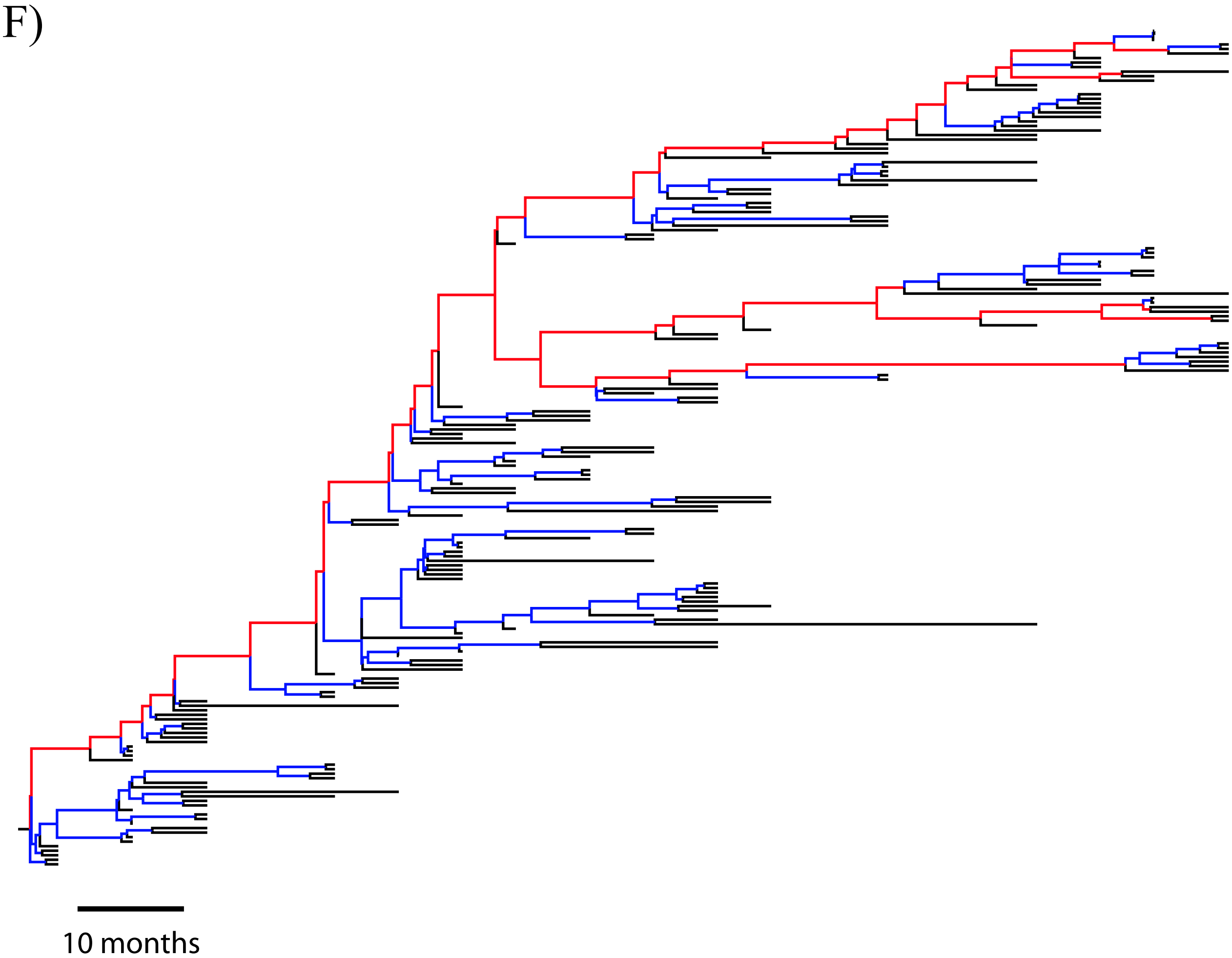

Figure S1

G)

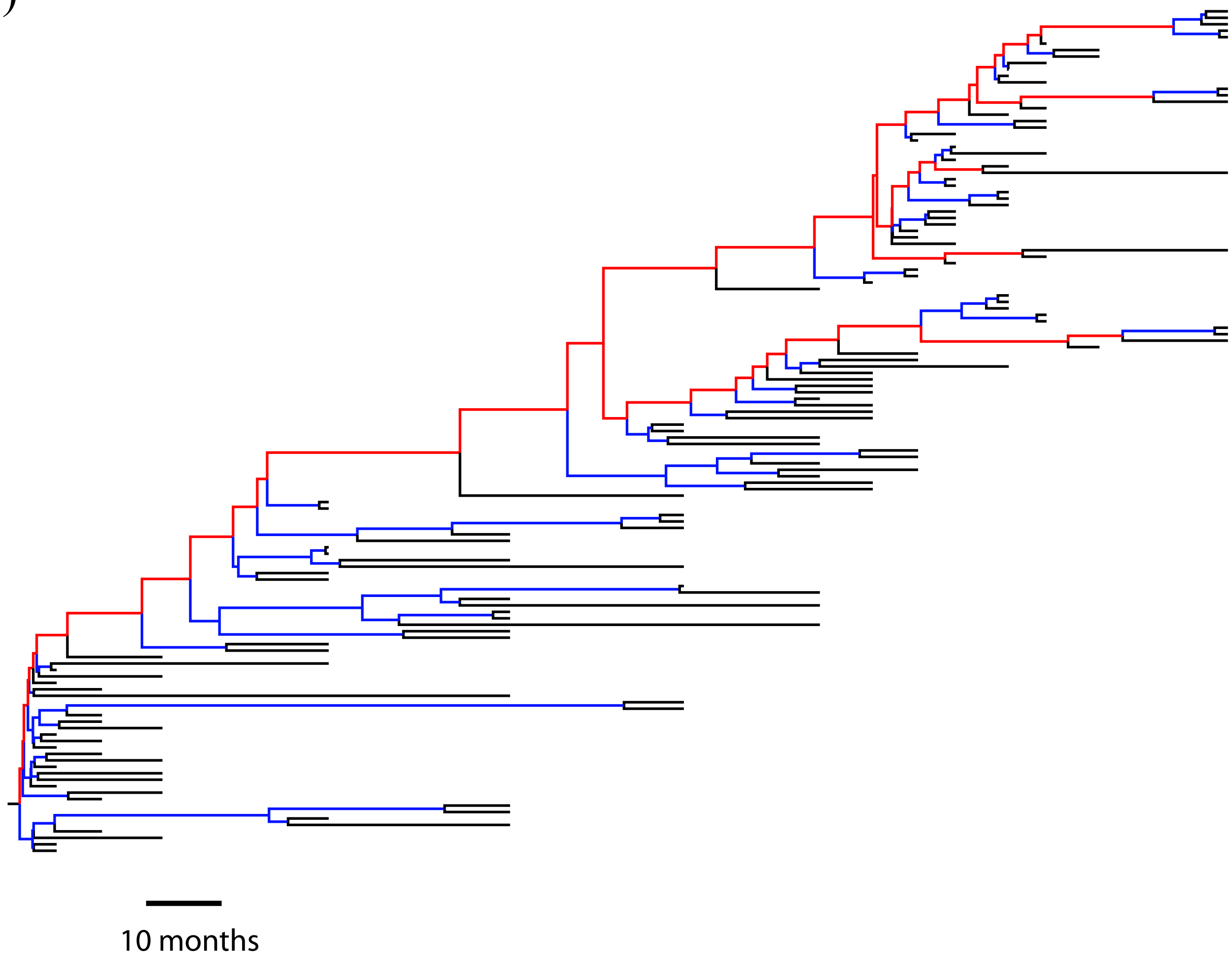

H)

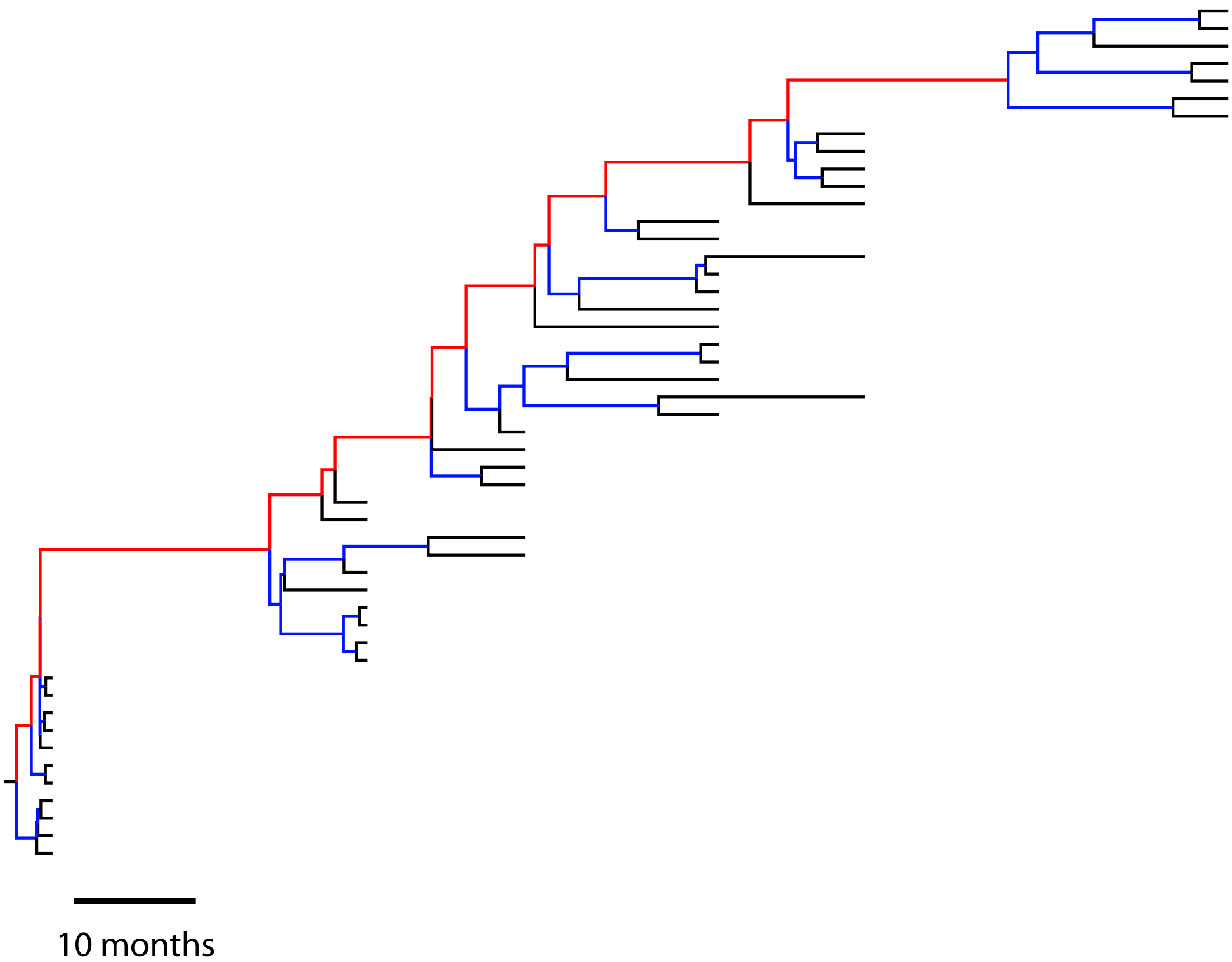

Supplement: Supplementary file 1 [file pcbi.0030029.sg001.pdf]
