# Supplementary material for: Synonymous Substitution Rates Predict HIV Disease Progression as a Result of Underlying Replication Dynamics
Source: PLoS Comput Biol. 2007 Feb 23;3(2):e29. doi: 10.1371/journal.pcbi.0030029 (PMC1797821; doi:10.1371/journal.pcbi.0030029)
Supplement: Supplementary file 2 [file pcbi.0030029.sg002.pdf]

Figure S2

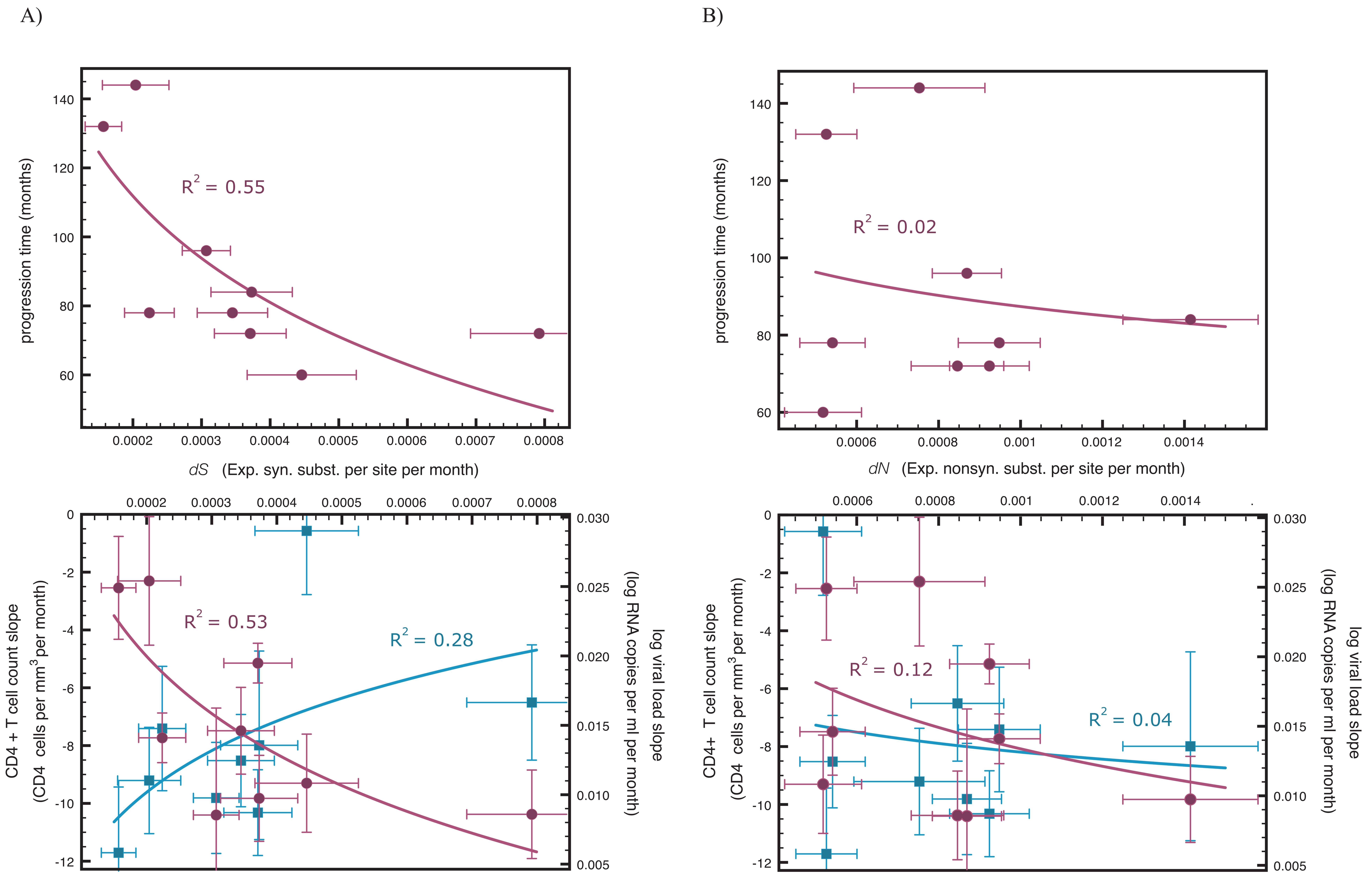
A)
B)
progression time (months)
140
120
100
80
60
R^2 = 0.55
0.0002
0.0003
0.0004
0.0005
0.0006
0.0007
0.0008
dS (Exp. syn. subst. per site per month)
R^2 = 0.02
0.0006
0.0008
0.001
0.0012
0.0014
dN (Exp. nonsyn. subst. per site per month)
CD4 + T cell count slope
(CD4 cells per mm^3 per month)
0
-2
-4
-6
-8
-10
-12
R^2 = 0.53
R^2 = 0.28
0.030
0.025
0.020
0.015
0.010
0.005
log viral load slope
(log RNA copies per ml per month)
CD4+ T cell count slope
R^2 = 0.12
R^2 = 0.04
