# Supplementary material for: Synonymous Substitution Rates Predict HIV Disease Progression as a Result of Underlying Replication Dynamics
Source: PLoS Comput Biol. 2007 Feb 23;3(2):e29. doi: 10.1371/journal.pcbi.0030029 (PMC1797821; doi:10.1371/journal.pcbi.0030029)
Supplement: Supplementary file 3 [file pcbi.0030029.sg003.pdf]

Figure S3

A) B)

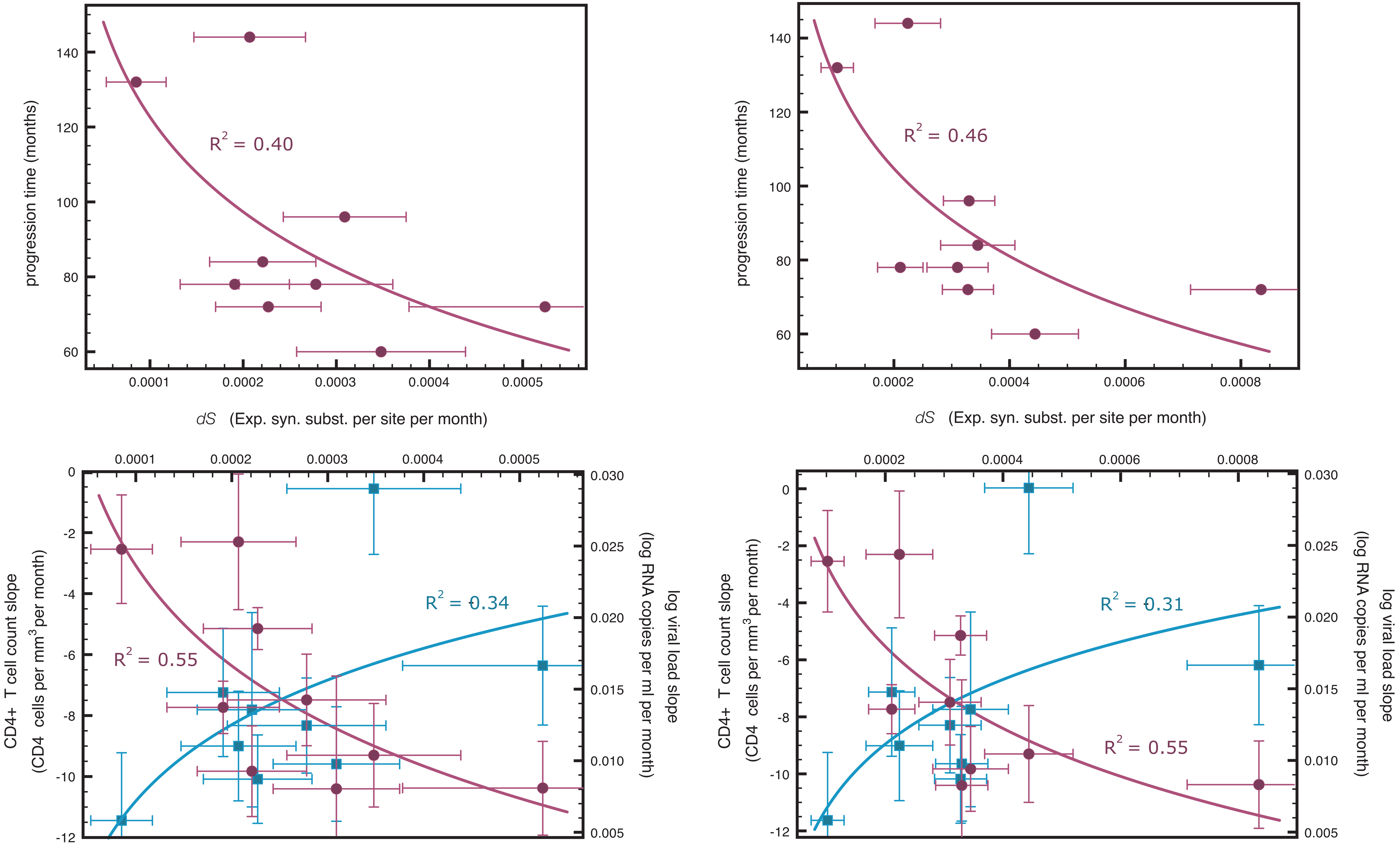

R² = 0.40
progression time (months)
60
80
100
120
140
0.0001
0.0002
0.0003
0.0004
0.0005
dS (Exp. syn. subst. per site per month)
R² = 0.46
progression time (months)
60
80
100
120
140
0.0002
0.0004
0.0006
0.0008
dS (Exp. syn. subst. per site per month)
0.0001
0.0002
0.0003
0.0004
0.0005
0
-2
-4
-6
-8
-10
-12
CD4+ T cell count slope
(CD4 cells per mm³ per month)
R² = 0.55
R² = 0.34
0.030
0.025
0.020
0.015
0.010
0.005
log viral load slope
(log RNA copies per ml per month)
0.0002
0.0004
0.0006
0.0008
0
-2
-4
-6
-8
-10
-12
CD4+ T cell count slope
(CD4 cells per mm³ per month)
R² = 0.31
R² = 0.55
0.030
0.025
0.020
0.015
0.010
0.005
log viral load slope
(log RNA copies per ml per month)
