# Supplementary material for: Synonymous Substitution Rates Predict HIV Disease Progression as a Result of Underlying Replication Dynamics
Source: PLoS Comput Biol. 2007 Feb 23;3(2):e29. doi: 10.1371/journal.pcbi.0030029 (PMC1797821; doi:10.1371/journal.pcbi.0030029)
Supplement: Supplementary file 4 [file pcbi.0030029.st001.doc]

**Table S1. Mean internal/external rate ratios for simulated and real data sets.**

|  | Patient 1 | Patient 2 | Patient 3 | Patient 5 | Patient 6 | Patient 7 | Patient 8 | Patient 9 | Patient 11 |
| --- | --- | --- | --- | --- | --- | --- | --- | --- | --- |
| Expo, CLOCK, 1 | 1.006 | 0.979 | 0.965 | 0.980 | 1.036 | 0.946 | 1.018 | 0.956 | 1.037 |
| Expo, CLOCK, 2 | 0.930 | 0.999 | 1.006 | 1.043 | 1.059 | 1.024 | 0.971 | 1.114 | 0.987 |
| Expo, CLOCK, 3 | 1.017 | 0.982 | 1.052 | 1.072 | 0.958 | 0.917 | 0.896 | 0.932 | 1.087 |
| Expo, CLOCK, 4 | 1.014 | 1.030 | 0.915 | 0.955 | 1.008 | 0.913 | 0.984 | 0.935 | 1.125 |
| Expo, CLOCK, 5 | 0.990 | 0.931 | 0.903 | 0.952 | 0.904 | 0.941 | 1.053 | 1.044 | 1.133 |
| Expo, UCED, 1 | 1.042 | 1.019 | 0.967 | 1.039 | 0.961 | 1.014 | 1.064 | 1.052 | 1.099 |
| Expo, UCED, 2 | 0.866 | 1.044 | 0.997 | 0.965 | 1.030 | 0.990 | 1.050 | 1.003 | 1.024 |
| Expo, UCED, 3 | 1.063 | 0.995 | 1.026 | 0.947 | 0.978 | 0.931 | 0.993 | 0.886 | 0.996 |
| Expo, UCED, 4 | 1.058 | 1.030 | 0.802 | 0.978 | 1.132 | 0.901 | 1.110 | 1.012 | 1.066 |
| Expo, UCED, 5 | 1.005 | 0.982 | 0.944 | 1.050 | 0.896 | 0.965 | 0.982 | 1.081 | 0.959 |
| Log, CLOCK, 1 | 1.013 | 1.006 | 0.974 | 0.992 | 0.916 | 1.032 | 1.028 | 0.982 | 1.007 |
| Log, CLOCK, 2 | 1.031 | 0.981 | 1.050 | 0.960 | 1.039 | 1.062 | 1.024 | 0.938 | 1.006 |
| Log, CLOCK, 3 | 1.041 | 1.002 | 0.987 | 1.013 | 0.986 | 1.038 | 1.020 | 0.941 | 1.114 |
| Log, CLOCK, 4 | 0.982 | 1.025 | 0.952 | 1.031 | 0.943 | 1.052 | 0.950 | 0.970 | 0.836 |
| Log, CLOCK, 5 | 1.051 | 1.016 | 1.115 | 1.022 | 0.934 | 1.115 | 0.963 | 1.077 | 1.008 |
| Log, UCED, 1 | 1.034 | 0.975 | 1.037 | 0.973 | 1.029 | 1.091 | 1.031 | 0.903 | 0.809 |
| Log, UCED, 2 | 1.105 | 1.068 | 1.075 | 0.976 | 1.087 | 1.145 | 0.938 | 1.003 | 1.283 |
| Log, UCED, 3 | 1.011 | 1.097 | 0.995 | 0.968 | 1.040 | 0.985 | 1.049 | 0.899 | 1.111 |
| Log, UCED, 4 | 0.839 | 1.013 | 0.960 | 0.929 | 1.034 | 0.885 | 0.954 | 0.874 | 0.950 |
| Log, UCED, 5 | 0.934 | 0.927 | 1.029 | 1.066 | 0.915 | 1.112 | 1.029 | 1.005 | 0.943 |
| Mean simulated | 1.002 | 1.005 | 0.988 | 0.996 | 0.994 | 1.003 | 1.005 | 0.980 | 1.029 |
| Real | 0.822 | 0.800 | 0.874 | 0.781 | 0.743 | 0.819 | 0.711 | 0.879 | 0.657 |
| *p* value | P < 0.05 | P < 0.05 | P = 0.05 | P < 0.05 | P < 0.05 | P < 0.05 | P < 0.05 | P = 0.05 | P < 0.05 |
