# Supplementary material for: Synonymous Substitution Rates Predict HIV Disease Progression as a Result of Underlying Replication Dynamics
Source: PLoS Comput Biol. 2007 Feb 23;3(2):e29. doi: 10.1371/journal.pcbi.0030029 (PMC1797821; doi:10.1371/journal.pcbi.0030029)
Supplement: Supplementary file 5 [file pcbi.0030029.st002.doc]

**Table S2A. Recombination rate estimates for each time point.**

|  | Patient1 | | | | Patient2 | | | | Patient3 | | | | Patient5 | | | | | Patient6 | | | | |
| --- | --- | --- | --- | --- | --- | --- | --- | --- | --- | --- | --- | --- | --- | --- | --- | --- | --- | --- | --- | --- | --- | --- |
| Tp | Ts | S | rho | SD | Ts | S | rho | SD | Ts | S | rho | SD | | Ts | S | rho | SD | | Ts | S | rho | SD |
| 1 | 3 | 7 | 0 | 1 | 5 | 27 | 2 | 36 | 4 | 6 | 0 | 10 | | 3 | 26 | 21 | 3 | | 3 | 20 | 12* | 7 |
| 2 | 14 | 20 | 8 | 36 | 12 | 19 | 0 | 12 | 9 | 8 | 12 | 12 | | 9 | 28 | 11* | 3 | | 9 | 11 | 32 | 20 |
| 3 | 24 | 30 | 2* | 6 | 20 | 43 | 40 | 20 | 26 | 26 | 45* | 13 | | 16 | 19 | 0 | 1 | | 18 | 25 | 17 | 9 |
| 4 | 34 | 23 | 22 | 32 | 30 | 67 | 100* | 30 | 30 | 29 | 0 | 4 | | 19 | 32 | 56* | 12 | | 24 | 38 | 51 | 19 |
| 5 | 45 | 36 | 48 | 18 | 40 | 36 | 5 | 3 | 42 | 28 | 2 | 1 | | 21 | 23 | 17* | 11 | | 30 | 26 | 49* | 13 |
| 6 | 51 | 42 | 4 | 2 | 51 | 78 | 93* | 11 | 55 | 48 | 76* | 13 | | 25 | 24 | 6 | 3 | | 36 | 37 | 34* | 8 |
| 7 | 61 | 42 | 42* | 6 | 61 | 78 | 22* | 3 | 67 | 57 | 6* | 3 | | 28 | 31 | 26 | 26 | | 42 | 28 | 8* | 21 |
| 8 | 66 | 35 | 4 | 1 | 68 | 55 | 47* | 14 | 73 | 61 | 15* | 4 | | 34 | 28 | 32 | 25 | | 48 | 31 | 39 | 22 |
| 9 | 68 | 28 | 12* | 11 | 73 | 76 | 70* | 10 | 80 | 50 | 4 | 2 | | 40 | 29 | 5* | 2 | | 54 | 33 | 39* | 11 |
| 10 | 77 | 32 | 29* | 15 | 80 | 19 | 9 | 8 | 85 | 54 | 7 | 1 | | 42 | 34 | 49* | 26 | | 73 | 60 | 65* | 20 |
| 11 | 80 | 25 | 6 | 3 | 85 | 74 | 22* | 3 | 91 | 51 | 27* | 2 | | 43 | 28 | 37* | 22 | | - | - | - | - |
| 12 | 87 | 27 | 26* | 3 | 91 | 39 | 15* | 4 | 96 | 27 | 0 | 0 | | 49 | 59 | 28* | 16 | | - | - | - | - |
| 13 | 94 | 17 | 6 | 4 | 103 | 63 | 29* | 2 | - | - | - | - | | 56 | 38 | 40* | 18 | | - | - | - | - |
| 14 | 98 | 13 | 17 | 20 | 126 | 53 | 37* | 6 | - | - | - | - | | 62 | 66 | 100* | 0 | | - | - | - | - |
| 15 | 105 | 36 | 31* | 16 | 138 | 29 | 23* | 4 | - | - | - | - | | 68 | 33 | 39 | 22 | | - | - | - | - |
| 16 | - | - | - | - | 144 | 35 | 0 | 1 | - | - | - | - | | 81 | 70 | 38* | 6 | | - | - | - | - |
| 17 | - | - | - | - | 158 | 62 | 6* | 1 | - | - | - | - | | - | - | - | - | | - | - | - | - |

**Table S2B. Recombination rate estimates for each time point.**

|  | Patient7 | | | | Patient8 | | | | Patient9 | | | | | Patient11 | | | |
| --- | --- | --- | --- | --- | --- | --- | --- | --- | --- | --- | --- | --- | --- | --- | --- | --- | --- |
| Tp | Ts | S | rho | SD | Ts | S | rho | SD | Ts | S | rho | SD | Ts | | S | rho | SD |
| 1 | 2 | 28 | 2 | 5 | 3 | 4 | 28 | 27 | 3 | 16 | 0 | 26 | 3 | | 21 | 0 | 3 |
| 2 | 25 | 40 | 7 | 6 | 10 | 18 | 4 | 25 | 9 | 19 | 0 | 1 | 29 | | 15 | 4 | 17 |
| 3 | 31 | 39 | 47 | 30 | 17 | 54 | 51 | 1 | 17 | 26 | 0 | 3 | 42 | | 21 | 0 | 2 |
| 4 | 44 | 83 | 35* | 3 | 29 | 29 | 3 | 5 | 39 | 42 | 30 | 9* | 58 | | 40 | 85* | 15 |
| 5 | 50 | 66 | 10* | 1 | 35 | 34 | 4 | 14 | 63 | 60 | 96 | 15* | 70 | | 59 | 100 | 18 |
| 6 | 56 | 56 | 11* | 2 | 41 | 60 | 62* | 4 | 86 | 58 | 21 | 2* | 100 | | 51 | 20 | 4 |
| 7 | 62 | 61 | 25* | 6 | 46 | 42 | 5 | 9 | 104 | 64 | 10 | 5* | - | | - | - | - |
| 8 | 74 | 83 | 8 | 2 | 53 | 48 | 27* | 2 | 111 | 59 | 32 | 5* | - | | - | - | - |
| 9 | 80 | 100 | 11* | 1 | 59 | 53 | 5* | 4 | 117 | 66 | 14 | 2* | - | | - | - | - |
| 10 | 87 | 94 | 3* | 0 | 65 | 87 | 23* | 5 | 122 | 27 | 41 | 14* | - | | - | - | - |
| 11 | - | - | - | - | 70 | 70 | 12* | 3 | 129 | 49 | 3 | 1* | - | | - | - | - |
| 12 | - | - | - | - | 81 | 59 | 19* | 12 | 134 | 53 | 10 | 3* | - | | - | - | - |
| 13 | - | - | - | - | 95 | 75 | 23* | 1 | 141 | 45 | 0 | 1 | - | | - | - | - |
| 14 | - | - | - | - | 101 | 47 | 0 | 14 | - | - | - | - | - | | - | - | - |
| 15 | - | - | - | - | 106 | 65 | 8* | 6 | - | - | - | - | - | | - | - | - |
