# Supplementary material for: Synonymous Substitution Rates Predict HIV Disease Progression as a Result of Underlying Replication Dynamics
Source: PLoS Comput Biol. 2007 Feb 23;3(2):e29. doi: 10.1371/journal.pcbi.0030029 (PMC1797821; doi:10.1371/journal.pcbi.0030029)
Supplement: Supplementary file 6 [file pcbi.0030029.st003.doc]

**Table S3. Analysis of among-site nonsynyonymous and synonymous rate variation**

| Patient | logL NS | logL Dual | p-value | CV (*dS*) | CV (*dN*) |
| --- | --- | --- | --- | --- | --- |
| P1 | -5210.7 | -4994.2 | <0.001 | 2.8 | 2.6 |
| P2 | -11677.0 | -11357.0 | <0.001 | 2.5 | 1.7 |
| P3 | -6696.8 | -6550.8 | <0.001 | 3.0 | 2.1 |
| P5 | -9290.0 | -8790.8 | <0.001 | 2.6 | 2.8 |
| P6 | -4810.9 | -4477.6 | <0.001 | 2.6 | 2.8 |
| P7 | -12921.8 | -12408.2 | <0.001 | 2.7 | 1.8 |
| P8 | -10820.4 | -10416.7 | <0.001 | 2.2 | 2.1 |
| P9 | -8274.9 | -7996.7 | <0.001 | 2.3 | 1.8 |
| P11 | -2717.5 | -2676.6 | <0.001 | 2.8 | 2.0 |
