# Supplementary material for: Synonymous Substitution Rates Predict HIV Disease Progression as a Result of Underlying Replication Dynamics
Source: PLoS Comput Biol. 2007 Feb 23;3(2):e29. doi: 10.1371/journal.pcbi.0030029 (PMC1797821; doi:10.1371/journal.pcbi.0030029)
Supplement: Supplementary file 7 [file pcbi.0030029.st004.doc]

**Table S4.** **Mean synonymous and nonsynonymous substitution rates for different HIV variants**

| Data set | | Internal rate (subst./site/month) | | Backbone rate (subst./site/month) | |
| --- | --- | --- | --- | --- | --- |
|  |  | synonymous | nonsynonymous | synonymous | nonsynonymous |
| HIV-1 group M | P6 | 7.28E-04 ± 1.49E-04 | 7.93E-04 ± 1.56E-04 | 7.97E-04 ± 2.25E-04 | 7.92E-04 ± 2.21E-04 |
|  | P5 | 1.03E-03 ± 1.57E-04 | 9.95E-04 ± 1.55E-04 | 7.27E-04 ± 1.82E-04 | 1.73E-03 ± 3.24E-04 |
|  | P8 | 3.95E-04 ± 7.03E-05 | 1.05E-03 ± 1.41E-04 | 3.37E-04 ± 1.06E-04 | 1.12E-03 ± 1.97E-04 |
|  | P1 | 3.79E-04 ± 7.75E-05 | 5.27E-04 ± 1.09E-04 | 3.51E-04 ± 1.23E-04 | 7.35E-04 ± 1.97E-04 |
|  | P7 | 2.84E-04 ± 5.21E-05 | 9.73E-04 ± 1.35E-04 | 3.19E-04 ± 9.02E-05 | 1.35E-03 ± 2.18E-04 |
|  | P3 | 5.53E-04 ± 1.09E-04 | 1.43E-04 ± 2.61E-04 | 2.98E-04 ± 1.17E-04 | 1.83E-03 ± 3.84E-04 |
|  | P2 | 4.12E-04 ± 5.95E-05 | 1.21E-03 ± 1.19E-04 | 4.43E-04 ± 1.33E-04 | 1.26E-03 ± 3.46E-04 |
|  | P9 | 1.44E-04 ± 2.55E-05 | 5.33E-04 ± 7.48E-05 | 1.75E-04 ± 4.31E-05 | 8.06E-04 ± 1.39E-04 |
|  | P11 | 3.26E-04 ± 8.51E-05 | 9.41E-04 ± 1.76E-04 | 2.86E-04 ± 1.48E-04 | 5.91E-04 ± 2.02E-04 |
| HIV-1 group O | Janssens | 5.17E-04 ± 7.82E-05 | 2.34E-03 ± 2.85E-04 | 4.80E-04 ± 9.75E-05 | 2.76E-03 ± 3.82E-04 |
|  | Chaix-Baudier | 7.75E-04 ± 2.64E-04 | 4.04E-03 ± 1.05E-03 | 5.76E-04 ± 3.05E-04 | 3.50E-03 ± 1.11E-03 |
| HIV-2 | Pc | 4.08E-04 ± 1.69E-04 | 8.63E-04 ± 3.29E-04 | 4.47E-04 ± 2.86E-04 | 3.79E-04 ± 2.87E-04 |
|  | Pd | 1.91E-04 ± 1.16E-04 | 7.23E-04 ± 3.91E-04 | 9.63E-05 ± 1.47E-04 | 5.08E-04 ± 3.90E-04 |
|  | P3P4 | 2.78E-04 ± 1.15E-04 | 1.89E-03 ± 7.15E-04 | 2.61E-04 ± 2.44E-04 | 1.44E-03 ± 1.14E-03 |
|  | P7P8 | 2.50E-04 ± 6.56E-05 | 3.76E-04 ± 7.74E-05 | 3.24E-04 ± 1.36E-04 | 4.26E-04 ± 1.65E-04 |
|  | P9P10 | 2.30E-04 ± 6.49E-05 | 2.88E-04 ± 5.27E-05 | 3.48E-04 ± 1.24E-04 | 2.76E-04 ± 7.03E-05 |
|  | Shi | 8.83E-05 ± 4.57E-05 | 1.71E-04 ± 7.11E-05 | 8.64E-05 ± 4.77E-05 | 2.01E-04 ± 8.32E-05 |
